# Supplementary material for: The coral pathogen Vibrio coralliilyticus uses a T6SS to secrete a group of novel anti-eukaryotic effectors that contribute to virulence
Source: PLoS Biol. 2024 Sep 3;22(9):e3002734. doi: 10.1371/journal.pbio.3002734 (PMC11371242; doi:10.1371/journal.pbio.3002734)
Supplement: S1 Text. Fig A — Comparison of T6SS1 clusters in the 31 V. coralliilyticus strains analyzed in this study. Colors denote homology between the encoded protein sequences. The strain name and RefSeq accession are denoted. Dashed, black vertical lines denote borders between separate contigs. Fig B. T6SS2 clusters share a similar synteny. Comparison of T6SS2 clusters in the 31 V. coralliilyticus strains analyzed in this study. Colors denote homology between the encoded protein sequences. The strain name and RefSeq accession are denoted. Dashed, black vertical lines denote borders between separate contigs. Fig C. Deletion of hcp1 or tssM2 inactivates T6SS1 or T6SS2, respectively. (A) Expression (cells) and secretion (media) of VgrG1 and Hcp2 from the indicated V. coralliilyticus strains grown for 4 h at 28°C in rich media (MLB). RNA polymerase sigma 70 (RNAp) was used as a loading and lysis control. Asterisks denote expected protein sizes. WT, wild-type. (B) The growth of the indicated V. coralliilyticus strains in MLB at 28°C measured as absorbance at 600 nm (OD600). Data are shown as the mean ± SD; n = 3. Results from a representative experiment out of at least 3 independent experiments are shown. The data underlying panel B of this figure can be found in S6 Data. Fig D. Vibrio coralliilyticus T6SS1 mediates interbacterial competition. (A, B) Viability counts (colony forming units; CFU) of V. alginolyticus 12G01 and V. campbellii ATCC 25920 prey strains before (0 h) and after (4 h) co-incubation with the indicated V. coralliilyticus OCN008 attacker strains on MLB plates at 28°C. The statistical significance between samples at the 4 h time point was calculated using an unpaired, two-tailed Student’s t test; WT, wild-type; DL, the assay’s detection limit. Data are shown as the mean ± SD; n = 3. The data shown are a representative experiment out of 3 independent experiments. The data underlying this figure can be found in S7 Data. Fig E. Non-structural proteins secreted by Vibrio coralliil [file pbio.3002734.s001.docx]

**Supplementary Information**

**The coral pathogen *Vibrio coralliilyticus* uses a T6SS to secrete a group of novel anti-eukaryotic effectors that contribute to virulence**

Supplementary Figures A-G

Supplementary Tables A-C

Supplementary References

**
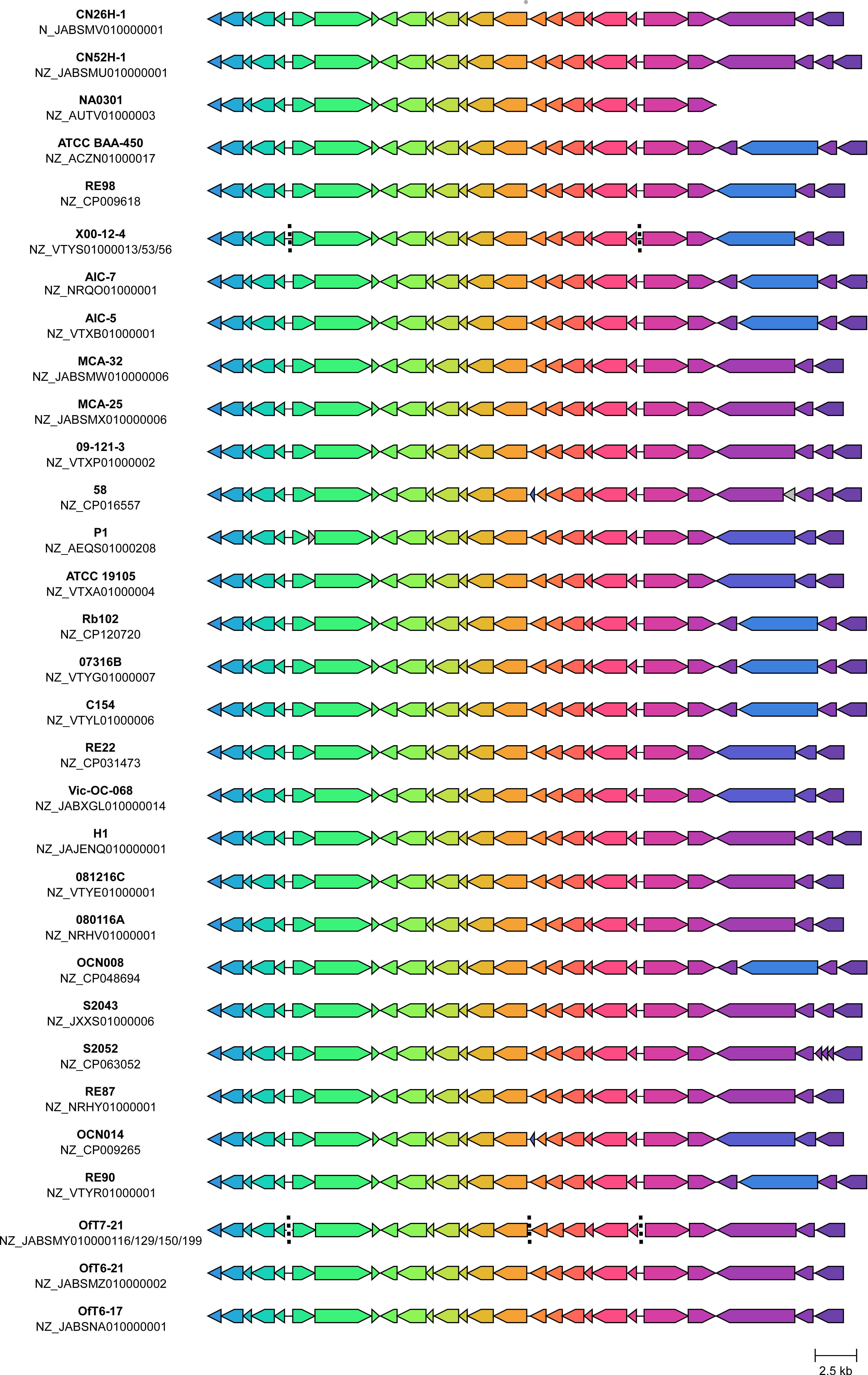
Supplementary Figures**

**Fig A. T6SS1 clusters share a similar synteny.** Comparison of T6SS1 clusters in the 31 *V. coralliilyticus* strains analyzed in this study. Colors denote homology between the encoded protein sequences. The strain name and RefSeq accession are denoted. Dashed, black vertical lines denote borders between separate contigs.

**
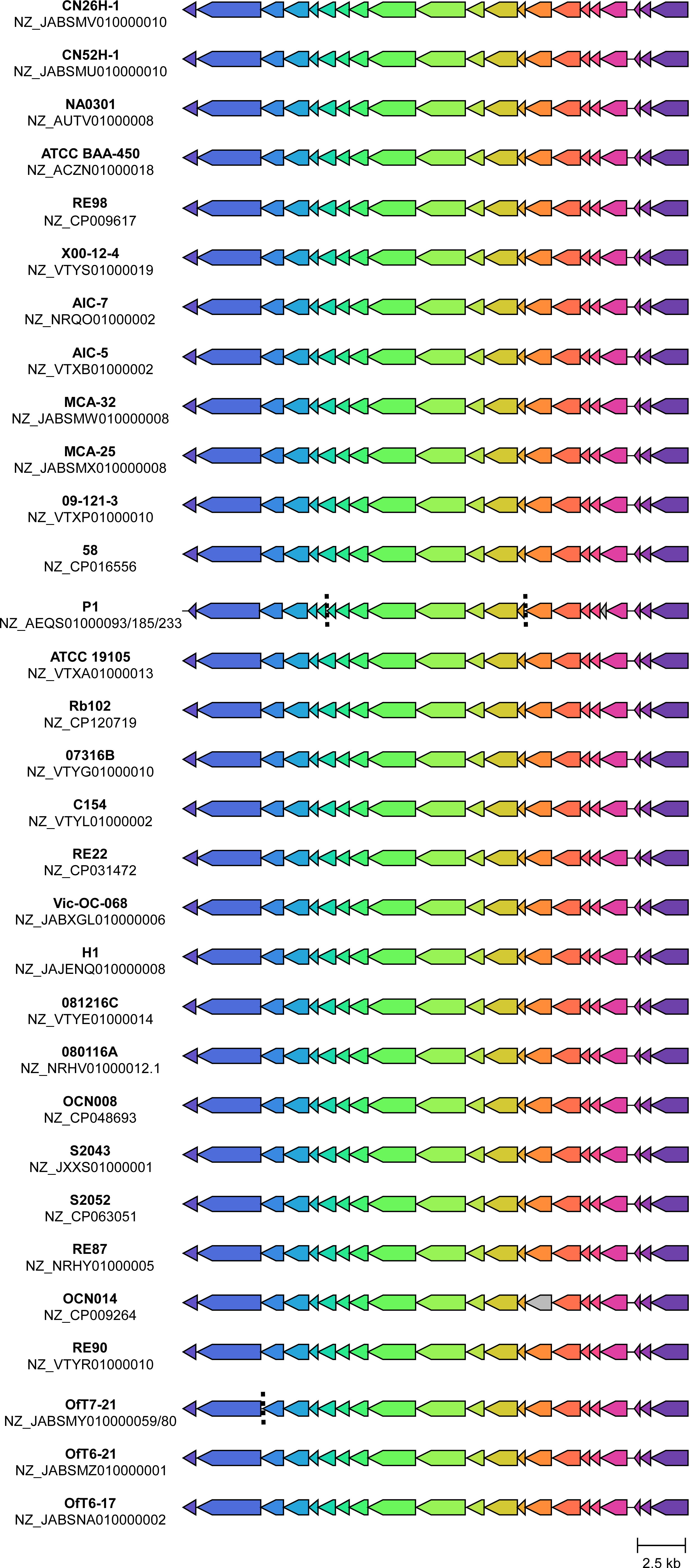
Fig B. T6SS2 clusters share a similar synteny.** Comparison of T6SS2 clusters in the 31 *V. coralliilyticus* strains analyzed in this study. Colors denote homology between the encoded protein sequences. The strain name and RefSeq accession are denoted. Dashed, black vertical lines denote borders between separate contigs.

**
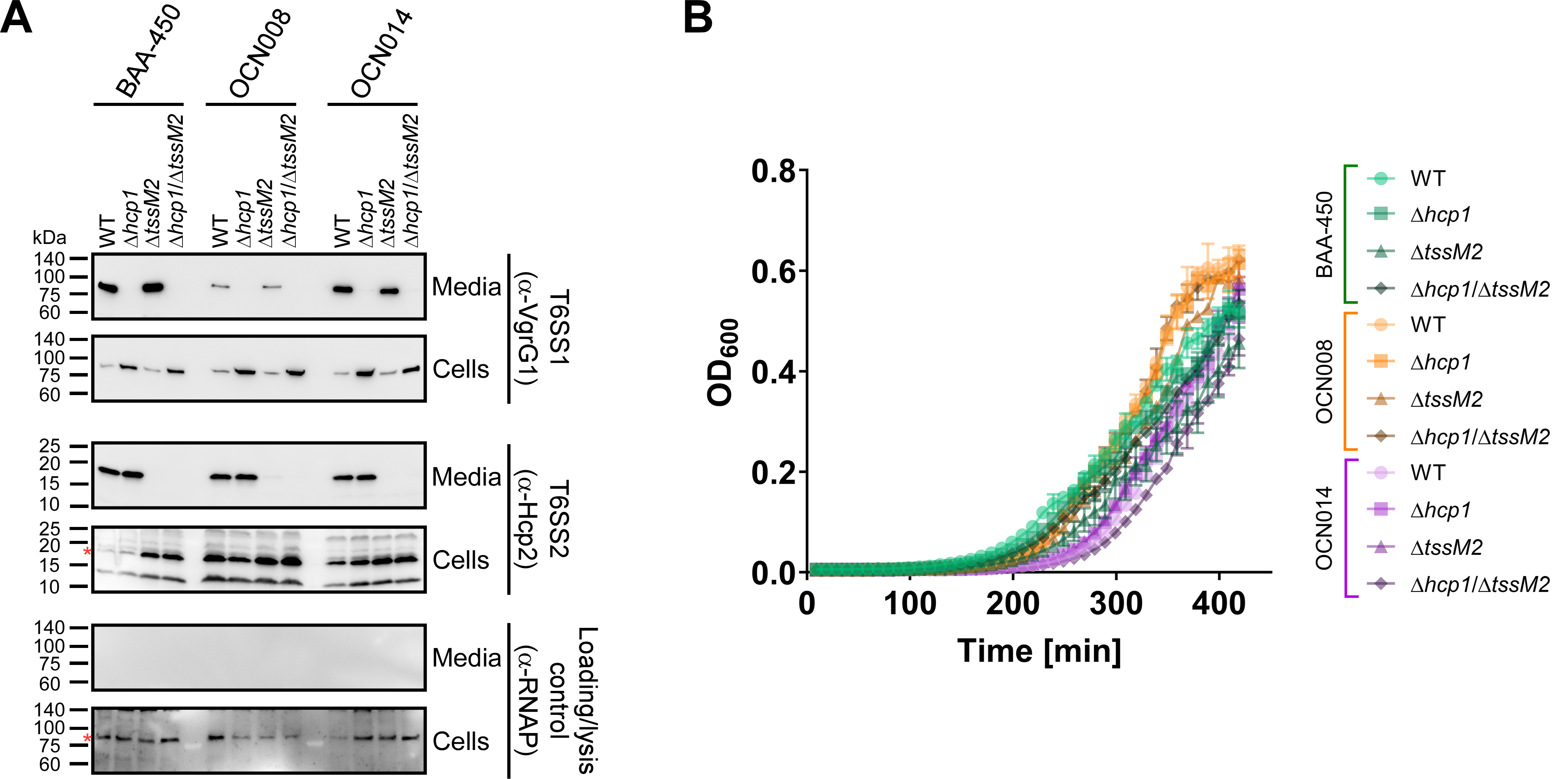
**

**Fig C. Deletion of *hcp1* or *tssM2* inactivates T6SS1 or T6SS2, respectively. A)** Expression (cells) and secretion (media) of VgrG1 and Hcp2 from the indicated *V. coralliilyticus* strains grown for 4 hours at 28°C in rich media (MLB). RNA polymerase sigma 70 (RNAp) was used as a loading and lysis control. Asterisks denote expected protein sizes. WT, wild-type. **B)** The growth of the indicated *V. coralliilyticus* strains in MLB at 28°C measured as absorbance at 600 nm (OD_600_). Data are shown as the mean ± SD; *n* = 3. Results from a representative experiment out of at least three independent experiments are shown. The data underlying panel B of this Figure can be found in S6 Data.

**
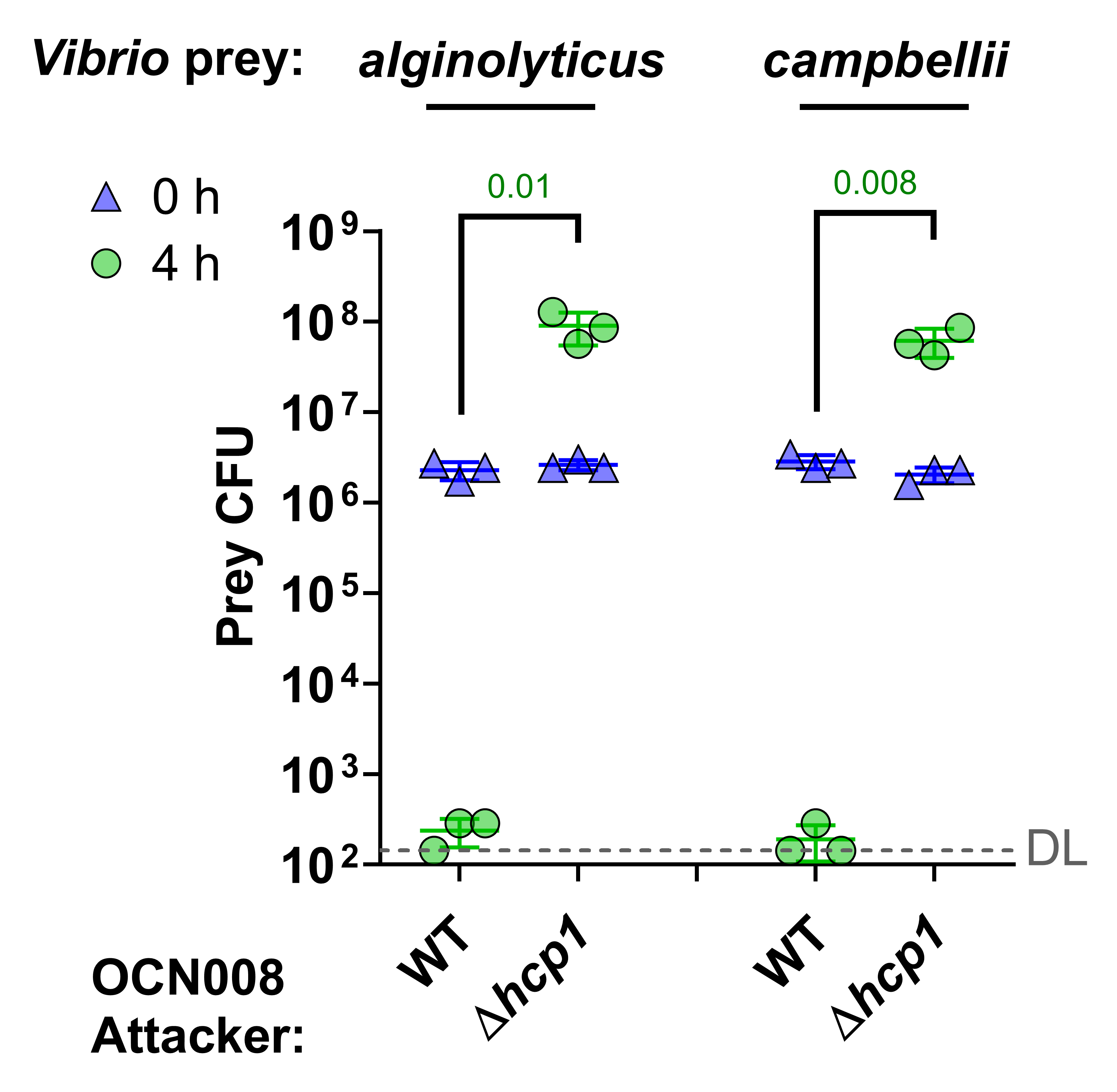
**

**Fig D. *Vibrio coralliilyticus* T6SS1 mediates interbacterial competition. A-B)** Viability counts (colony forming units; CFU) of *V. alginolyticus* 12G01 and *V. campbellii* ATCC 25920 prey strains before (0 h) and after (4 h) co-incubation with the indicated *V. coralliilyticus* OCN008 attacker strains on MLB plates at 28°C. The statistical significance between samples at the 4 h time point was calculated using an unpaired, two-tailed Student’s *t* test; WT, wild-type; DL, the assay’s detection limit. Data are shown as the mean ± SD; *n* = 3. The data shown are a representative experiment out of three independent experiments. The data underlying this Figure can be found in S7 Data.

**
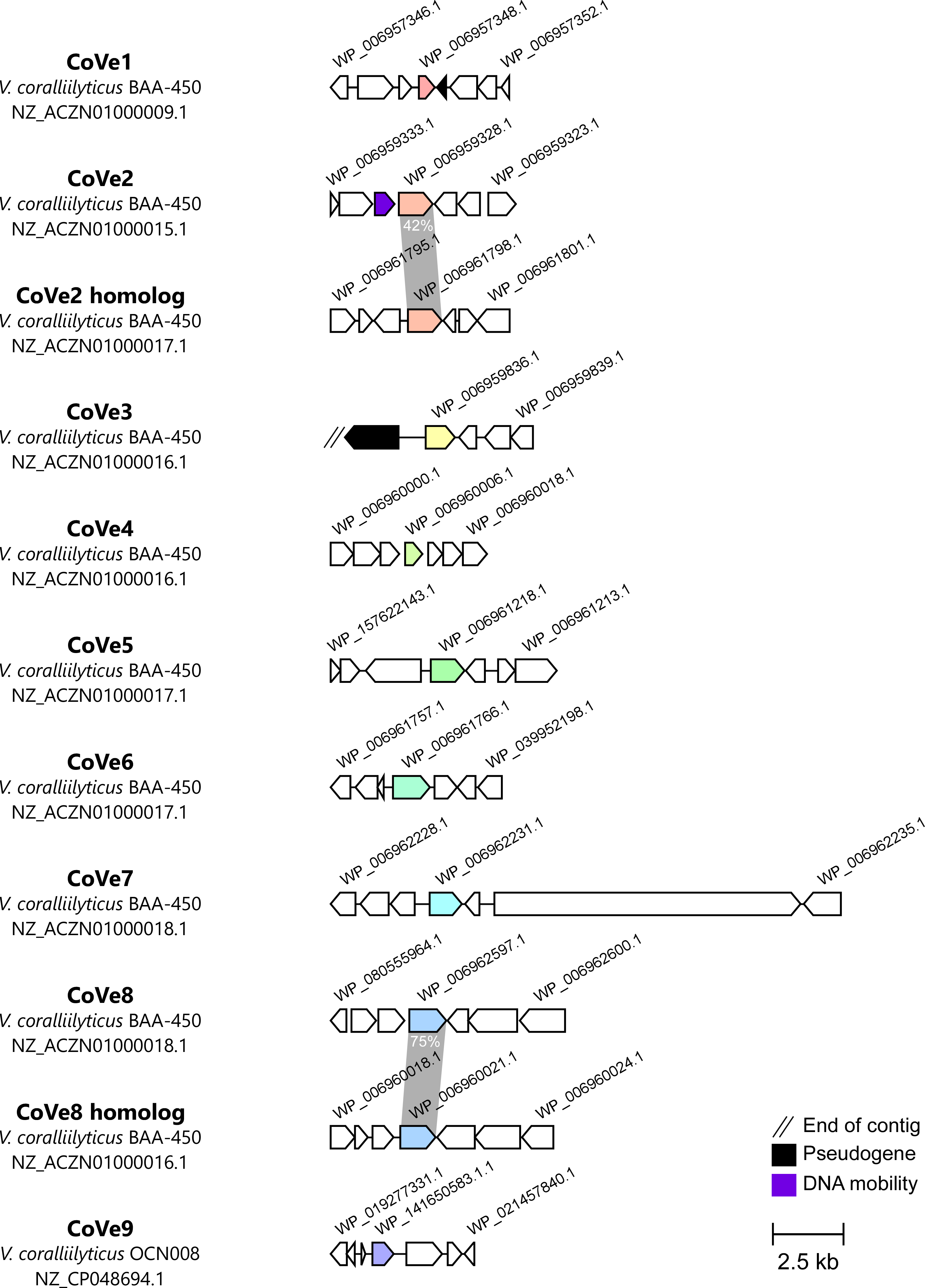
**

**Fig E. Non-structural proteins secreted by *Vibrio coralliilyticus* T6SS2 are encoded by orphan genes.** Genomic neighborhoods of genes encoding representative T6SS effector proteins (CoVes) and their homologs (colored arrows). The strain names, the GenBank accession numbers, and protein accessions are denoted. Genes are denoted by arrows indicating the predicted direction of transcription. Gray rectangles denote regions of amino acid sequence homology; identity percentages are indicated.


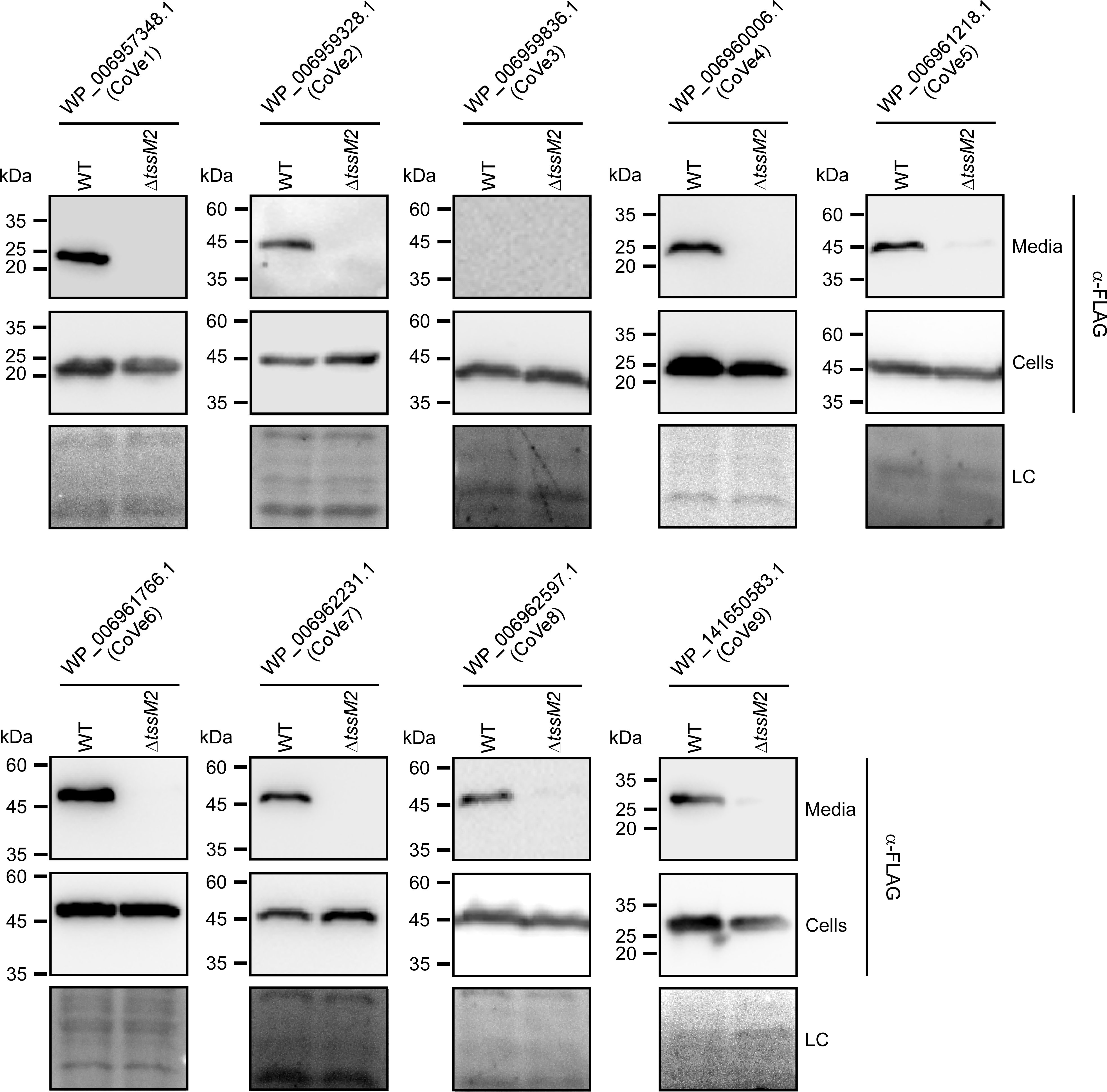


**Fig F. CoVes are secreted in a T6SS2-dependent manner.** Expression (cells) and secretion (media) of C-terminally FLAG-tagged CoVes expressed from arabinose-inducible plasmids in *V. coralliilyticus* strains, either wild-type (WT) or T6SS2^−^ (∆*tssM2*). CoVe1-8 were monitored in *V. coralliilyticus* BAA-450 and CoVe9 was monitored in *V. coralliilyticus* OCN008. *V. coralliilyticus* strains were grown in MLB supplemented with chloramphenicol and 0.01% (wt/vol) L-arabinose for 4 hours at 28°C. Loading control (LC) is shown for total protein lysate. Results from a representative experiment out of at least two independent experiments are shown.


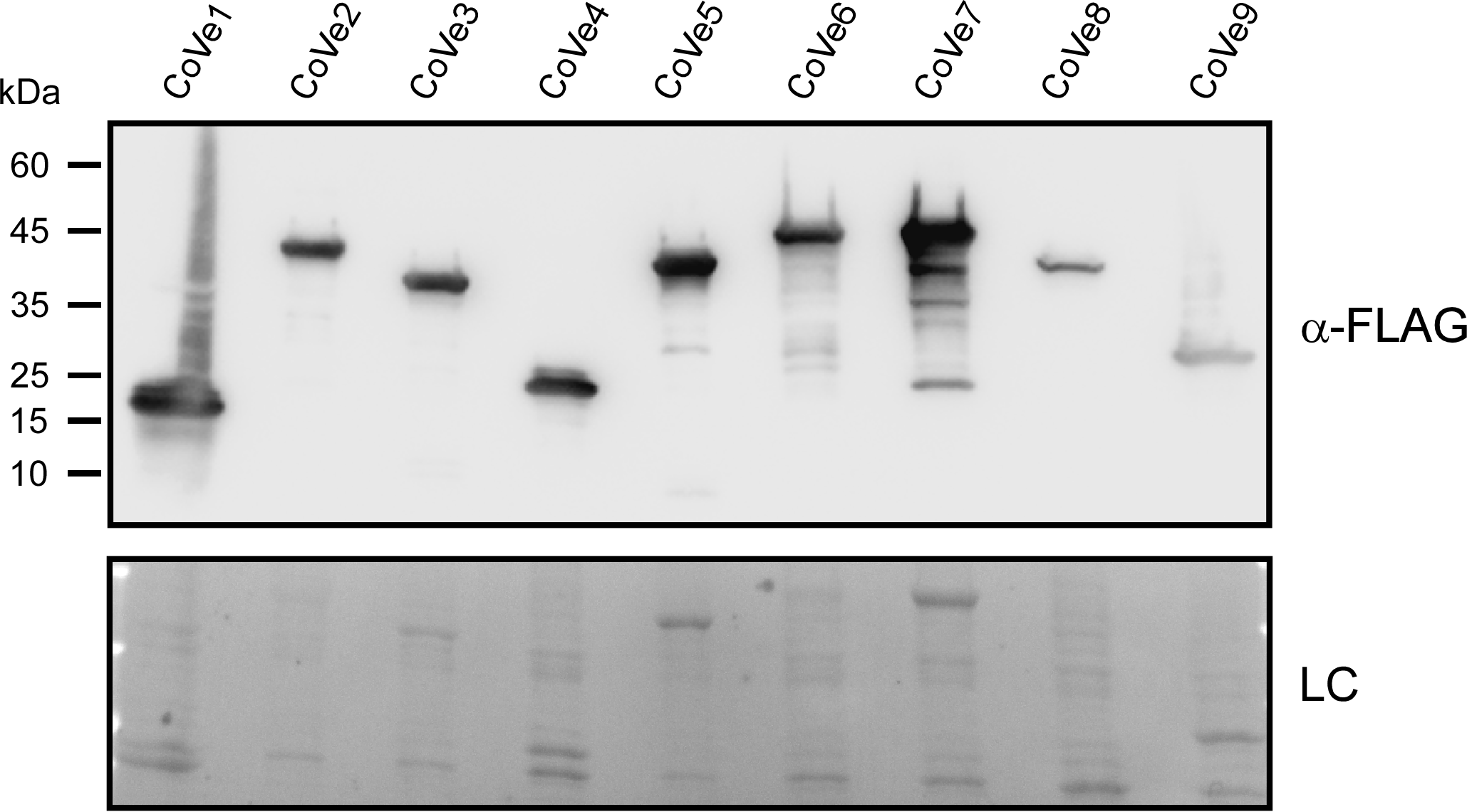


**Fig G. CoVes are expressed in *E. coli*.** Expression of C-terminally FLAG-tagged CoVes from arabinose-inducible plasmids in *E. coli* strain DH5α (λ-pir). Loading control (LC) is shown for total protein lysate. Results from a representative experiment out of at least two independent experiments are shown.

**Supplementary Tables**

**Table A. Bacteria and yeast strains used in this study.**

| Strain name | Genotype | Comments | Source |
| --- | --- | --- | --- |
| *Vibrio coralliilyticus* BAA-450 | Wild-type | Used for generating deletion strains, as an attacker in competition assays, in BMDM infection assays, and in secretion assays | [1] |
| *Vibrio coralliilyticus* BAA-450 ∆*hcp1* | ∆*VIC_RS16330* | BAA-450 derivative containing a deletion in *hcp1*; used as an attacker in competition assays, in BMDM infection assays, and in secretion assays | [2] |
| *Vibrio coralliilyticus* BAA-450 ∆*tssM2* | ∆*VIC_RS20055* | BAA-450 derivative containing a deletion in *tssM2*; used as an attacker in competition assays, in BMDM infection assays, and in secretion assays | This study |
| *Vibrio coralliilyticus* BAA-450 ∆*hcp1*/∆*tssM2* | ∆*VIC_RS16330*/∆*VIC_RS20055* | BAA-450 derivative containing a deletion in *hcp1 and in tssM2*; used as an attacker in competition assays, in BMDM infection assays, and in secretion assays | This study |
| *Vibrio coralliilyticus* OCN008 | Wild-type | Used for generating deletion strains, as an attacker in competition assays, in BMDM infection assays, in *Artemia* infection assays, and in secretion assays | [3] |
| *Vibrio coralliilyticus* OCN008 ∆*hcp1* | ∆*G3U99_RS23805* | OCN008 derivative containing a deletion in *hcp1*; used as an attacker in competition assays, in BMDM infection assays, in *Artemia* infection assays, and in secretion assays | This study |
| *Vibrio coralliilyticus* OCN008 ∆*tssM2* | ∆*G3U99_13155* | OCN008 derivative containing a deletion in *tssM2*; used as an attacker in competition assays, in BMDM infection assays, in *Artemia* infection assays, and in secretion assays | This study |
| *Vibrio coralliilyticus* OCN008 ∆*hcp1*/∆*tssM2* | ∆*G3U99_RS23805*/∆*G3U99_13155* | OCN008 derivative containing a deletion in *hcp1* and in *tssM2*; used as an attacker in competition assays, in BMDM infection assays, in *Artemia* infection assays, and in secretion assays | This study |
| *Vibrio coralliilyticus* OCN014 | Wild-type | Used for generating deletion strains, as an attacker in competition assays, in BMDM infection assays, and in secretion assays | [4] |
| *Vibrio coralliilyticus* OCN014 ∆*hcp1* | ∆*JV59_RS20030* | OCN014 derivative containing a deletion in *hcp1*; used as an attacker in competition assays, in BMDM infection assays, and in secretion assays | This study |
| *Vibrio coralliilyticus* OCN014 ∆*tssM2* | ∆*JV59_31975* | OCN014 derivative containing a deletion in *tssM2*; used as an attacker in competition assays, in BMDM infection assays, and in secretion assays | This study |
| *Vibrio coralliilyticus* OCN014 ∆*hcp1*/∆*tssM2* | ∆*JV59_RS20030*/∆*JV59_31975* | OCN014 derivative containing a deletion in *hcp1 and in tssM2*; used as an attacker in competition assays, in BMDM infection assays, and in secretion assays | This study |
| *Vibrio alginolyticus* 12G01 | Wild-type | Used as prey in competition assays | Lab stocks |
| *Vibrio campbellii* ATCC 25920 | Wild-type | Used as prey in competition assays | ATCC |
| *Vibrio natriegens* ATCC 14048 | Wild-type | Used as prey in competition assays | ATCC |
| *Escherichia coli* DH5α (λ-pir) | K-12 derivative laboratory strain containing λ-pir | Used for plasmid maintenance, cloning, protein expression, and toxicity assays | Gift from Prof. Eric V. Stabb |
| *Saccharomyces cerevisiae* BY4741 | MATa his3Δ1 leu2Δ0 met15Δ0 ura3Δ0 | A yeast strain used for protein expression and toxicity assays | Lab stocks |

**Table B. Plasmids used in this study.**

| Plasmid name | Description | Purpose | Source |
| --- | --- | --- | --- |
| pBAD33.1 | A mobilizable plasmid for arabinose-inducible protein expression, with ori15A and chloramphenicol resistance. | Used for selective growth of *V. alginolyticus* and *V. natriegens* prey. | [5] |
| pBAD33.1^F^ | pBAD33.1 with a FLAG tag inserted at the 3' end of the MCS | Used for the arabinose-inducible expression of proteins | [6] |
| psfGFP | pBAD33.1^F^ plasmid containing the CDS of sfGFP in-frame with the C-terminal FLAG tag | Used to construct the pKara1 plasmid | [7] |
| pWP_006957348.1 | pBAD33.1^F^ plasmid containing the CDS of WP_006957348.1 from *V. coralliilyticus* BAA-450 in-frame with the C-terminal FLAG tag of the plasmid | Used for the arabinose-inducible expression in *E. coli* and *V. coralliilyticus* | This study |
| pWP_006959328.1 | pBAD33.1^F^ plasmid containing the CDS of WP_006959328.1 from *V. coralliilyticus* BAA-450 in-frame with the C-terminal FLAG tag of the plasmid | Used for the arabinose-inducible expression in *E. coli* and *V. coralliilyticus* | This study |
| pWP_006960006.1 | pBAD33.1^F^ plasmid containing the CDS of WP_006960006.1 from *V. coralliilyticus* BAA-450 in-frame with the C-terminal FLAG tag of the plasmid | Used for the arabinose-inducible expression in *E. coli* and *V. coralliilyticus* | This study |
| pWP_006961766.1 | pBAD33.1^F^ plasmid containing the CDS of WP_006961766.1 from *V. coralliilyticus* BAA-450 in-frame with the C-terminal FLAG tag of the plasmid | Used for the arabinose-inducible expression in *E. coli* and *V. coralliilyticus* | This study |
| pWP_006962231.1 | pBAD33.1^F^ plasmid containing the CDS of WP_006962231.1 from *V. coralliilyticus* BAA-450 in-frame with the C-terminal FLAG tag of the plasmid | Used for the arabinose-inducible expression in *E. coli* and *V. coralliilyticus* | This study |
| pWP_141650583.1 | pBAD33.1^F^ plasmid containing the CDS of WP_141650583.1 from *V. coralliilyticus* OCN008 in-frame with the C-terminal FLAG tag of the plasmid | Used for the arabinose-inducible expression in *E. coli* and *V. coralliilyticus* | This study |
| pWP_006959836.1 | pBAD33.1^F^ plasmid containing the CDS of WP_006959836.1 from *V. coralliilyticus* OCN008 in-frame with the C-terminal FLAG tag of the plasmid | Used for the arabinose-inducible expression in *E. coli* | This study |
| p WP_006961218.1 | pBAD33.1^F^ plasmid containing the CDS of WP_006961218.1 from *V. coralliilyticus* OCN008 in-frame with the C-terminal FLAG tag of the plasmid | Used for the arabinose-inducible expression in *E. coli* | This study |
| pWP_006962597.1 | pBAD33.1^F^ plasmid containing the CDS of WP_006962597.1 from *V. coralliilyticus* OCN008 in-frame with the C-terminal FLAG tag of the plasmid | Used for the arabinose-inducible expression in *E. coli* | This study |
| pVSV208 | deRed and Cm^R^ cassette-containing plasmid | Used as the backbone to construct the pKara1 plasmid, and to selectively grow *V. campbellii* prey | [8] |
| pKara1 | pVSV208 containing the araC cassette amplified from psfGFP, upstream of the dsRed gene | Used for the arabinose-inducible expression | This study |
| pKara1:WP_006959836.1 | pKara1 plasmid containing the CDS of WP_006959836.1 from *V. coralliilyticus* BAA-450 replacing sfGFP, in-frame with the C-terminal FLAG tag of the plasmid | Used for the arabinose inducible expression in *V. coralliilyticus* | This study |
| pKara1:WP_006961218.1 | pKara1 plasmid containing the CDS of WP_006961218.1 from *V. coralliilyticus* BAA-450 replacing sfGFP, in-frame with the C-terminal FLAG tag of the plasmid | Used for the arabinose inducible expression in *V. coralliilyticus* | This study |
| pKara1:WP_006962597.1 | pKara1 plasmid containing the CDS of WP_006962597.1 from *V. coralliilyticus* BAA-450 replacing sfGFP, in-frame with the C-terminal FLAG tag of the plasmid | Used for the arabinose inducible expression in *V. coralliilyticus* | This study |
| pGML10 | pGML10 *E. coli*-*S. cerevisiae* shuttle vector, GAL1-10 promoter with a Myc tag at the 3' end of the MCS | Used for galactose-inducible expression in *S. cerevisiae* | Riken |
| pGML10:eGFP | pGML10 containing the CDS of enhanced GFP in the *EcoRI* site of the MCS, in-frame with the C -terminal Myc tag of the plasmid | Used for galactose-inducible expression in *S. cerevisiae* | [2] |
| pGML10:WP_006957348.1 | pGML10 containing the CDS of WP_006957348.1 from *V. coralliilyticus* BAA-450 in-frame with the C-terminal Myc tag of the plasmid | Used for galactose-inducible expression in *S. cerevisiae* | This study |
| pGML10:WP_006959328.1 | pGML10 containing the CDS of WP_006959328.1 from *V. coralliilyticus* BAA-450 in-frame with the C-terminal Myc tag of the plasmid | Used for galactose-inducible expression in *S. cerevisiae* | This study |
| pGML10:WP_006959836.1 | pGML10 containing the CDS of WP_006959836.1 from *V. coralliilyticus* BAA-450 in-frame with the C-terminal Myc tag of the plasmid | Used for galactose-inducible expression in *S. cerevisiae* | This study |
| pGML10:WP_006960006.1 | pGML10 containing the CDS of WP_006960006.1 from *V. coralliilyticus* BAA-450 in-frame with the C-terminal Myc tag of the plasmid | Used for galactose-inducible expression in *S. cerevisiae* | This study |
| pGML10:WP_006961218.1 | pGML10 containing the CDS of WP_006961218.1 from *V. coralliilyticus* BAA-450 in-frame with the C-terminal Myc tag of the plasmid | Used for galactose-inducible expression in *S. cerevisiae* | This study |
| pGML10:WP_006961766.1 | pGML10 containing the CDS of WP_006961766.1 from *V. coralliilyticus* BAA-450 in-frame with the C-terminal Myc tag of the plasmid | Used for galactose-inducible expression in *S. cerevisiae* | This study |
| pGML10:WP_006962231.1 | pGML10 containing the CDS of WP_006962231.1 from *V. coralliilyticus* BAA-450 in-frame with the C-terminal Myc tag of the plasmid | Used for galactose-inducible expression in *S. cerevisiae* | This study |
| pGML10:WP_006962597.1 | pGML10 containing the CDS of WP_006962597.1 from *V. coralliilyticus* BAA-450 in-frame with the C-terminal Myc tag of the plasmid | Used for galactose-inducible expression in *S. cerevisiae* | This study |
| pGML10:WP_141650583.1 | pGML10 containing the CDS of WP_141650583.1 from *V. coralliilyticus* BAA-450 in-frame with the C-terminal Myc tag of the plasmid | Used for galactose-inducible expression in *S. cerevisiae* | This study |
| pDM4 | a Cm^R^ and ori_R6K_-containing suicide vector | Used to generate deletions in *V. coralliilyticus* genomes | [9] |
| pDM4:*tssM2*^BAA-450^ | pDM4 containing 1 kb downstream and 1 kb upstream of *VIC_RS16330* in its MCS | Used to delete *tssM2* in *V. coralliilyticus* BAA-450 | This study |
| pDM4:*hcp1*^OCN008^ | pDM4 containing 1 kb downstream and 1 kb upstream of *G3U99_RS23805* in its MCS | Used to delete *hcp1* in *V. coralliilyticus* OCN008 | This study |
| pDM4:*tssM2*^OCN008^ | pDM4 containing 1 kb downstream and 1 kb upstream of *G3U99_13155* in its MCS | Used to delete *tssM2* in *V. coralliilyticus* OCN008 | This study |
| pDM4:*hcp1*^OCN014^ | pDM4 containing 1 kb downstream and 1 kb upstream of *JV59_RS20030* in its MCS | Used to delete *hcp1* in *V. coralliilyticus* OCN014 | This study |
| pDM4:*tssM2*^OCN014^ | pDM4 containing 1 kb downstream and 1 kb upstream of *JV59_31975* in its MCS | Used to delete *tssM2* in *V. coralliilyticus* OCN014 | This study |

**Table C. Primers used in this study.**

|  |  |  |
| --- | --- | --- |
|  |  |  |
|  |  |  |
|  |  |  |
|  |  |  |
|  |  |  |
|  |  |  |
|  |  |  |
| Top of Form | | |

| Primer name | Sequence (5' to 3')^#^ | Description |
| --- | --- | --- |
| \| pDM4_SacI_Gib_R \| \| --- \| \|  \| | GAGCTCTCCCGGGATTCCACAAATTG | Used to amplify the pDM4 plasmid backbone for Gibson assembly |
| \| pDM4_Sall_Gib_F \| \| --- \| | GTCGACGGTATCGATAAGCTTGATATACAC |  |
| OCN008_hcp1_Gib_UP_F | GAATCCCGGGAGAGCTCtgaatttactcag | Used to amplify 1 kb upstream of *G3U99_RS23805* to construct pDM4:*hcp1*^OCN008^ |
| OCN008_hcp1_Gib_UP_R | cccttagcgttcggtaagaaaatccttatattccttctaaaaaatttc |  |
| OCN008_hcp1_Gib_DN_F | gaaattttttagaaggaatataaggattttcttaccgaacgctaaggg | Used to amplify 1 kb downstream of *G3U99_RS23805* to construct pDM4:*hcp1*^OCN008^ |
| OCN008_hcp1_Gib_DN_R | GCTTATCGATACCGTCGACtcaccaatattaaagctg |  |
| OCN008/014_hcp1_F_ForPCR | atgccaactcctgcgtatatg | Used to validate deletion of *G3U99_RS23805* from *V. coralliilyticus* OCN008 (with OCN008_hcp1_R_ForPCR) and *JV59_RS20030* from *Vibrio coralliilyticus* OCN014 (with OCN014_hcp1_R_ForPCR) |
| OCN008_hcp1_R_ForPCR | cctttacccattacttcgtg | Used to validate deletion of *G3U99_RS23805* from *V. coralliilyticus* OCN008 (with OCN008/014_hcp1_F_ForPCR) |
| OCN014_hcp1_Gib_UP_F | GAATCCCGGGAGAGCTCtgaatttactcagcg | Used to amplify 1 kb upstream of *JV59_RS20030* to construct pDM4:*hcp1*^OCN014^ |
| OCN014_hcp1_Gib_UP_R | ccttagcgttcggtaagaaaatccttatattccttctaaaaaatttc |  |
| OCN014_hcp1_Gib_DN_F | gaaattttttagaaggaatataaggattttcttaccgaacgctaagg | Used to amplify 1 kb downstream of *JV59_RS20030* to construct pDM4:*hcp1*^OCN014^ |
| OCN014_hcp1_Gib_DN_R | GCTTATCGATACCGTCGACtcaccaatattaaagctgtaac |  |
| OCN014_hcp1_R_ForPCR | ttcgattgcacggtaagtg | Used to validate deletion of *JV59_RS20030* from *V. coralliilyticus* OCN014 (with OCN008/014_hcp1_F_ForPCR) |
| BAA450_tssM2_Gib_UP_F_(SM) | GAATCCCGGGAGAGCTCtaaattaaagg | Used to amplify 1 kb upstream of *VIC_RS16330* to construct pDM4:*tssM2*^BAA-450^ |
| BAA450_tssM2_Gib_UP_R_(SM) | cttcagaggctccttaggaatgtgaagataaaccgcgaag |  |
| BAA450_tssM2_Gib_DN_F_(SM) | cttcgcggtttatcttcacattcctaaggagcctctgaag | Used to amplify 1 kb downstream of *VIC_RS16330* to construct pDM4:*tssM2*^BAA-450^ |
| BAA450_tssM2_Gib_DN_R_(SM) | GCTTATCGATACCGTCGACtgccagaaccagaag |  |
| OCN008_tssM2_Gib_UP_F_(SM) | GAATCCCGGGAGAGCTCtgccagaacc | Used to amplify 1 kb upstream of *G3U99_13155* to construct pDM4:*tssM2*^OCN008^ |
| OCN008_tssM2_Gib_UP_R_(SM) | cgcggtttatcttcacattcctaaggagcctct |  |
| OCN008_tssM2_Gib_DN_F_(SM) | agaggctccttaggaatgtgaagataaaccgcg | Used to amplify 1 kb downstream of *G3U99_13155* from *V. coralliilyticus* OCN008 and *JV59_31975* from *V. coralliilyticus* OCN014 to construct pDM4:*tssM2*^OCN008^ and pDM4:*tssM2*^OCN014^ |
| OCN008/014_tssM2_Gib_DN_R_(SM) | GCTTATCGATACCGTCGACtaaattaaaggcggaag |  |
| OCN014_tssM2_Gib_UP_F_(SM) | GAATCCCGGGAGAGCTCaaccagaagatgtcac | Used to amplify 1 kb upstream of *JV59_31975* to construct pDM4:*tssM2*^OCN014^ |
| OCN014_tssM2_Gib_UP_R_(SM) | ggtttatcttcacattcctaaggagcctctga |  |
| OCN014_tssM2_Gib_DN_F_(SM) | tcagaggctccttaggaatgtgaagataaacc | Used to amplify 1 kb downstream of *JV59_31975* to construct pDM4:*tssM2*^OCN014^ |
| tssM2_F_ForPCR | ttagatactcgatggtaatgagaaggcag | Used to validate deletion of *VIC_RS16330* from *V. coralliilyticus* BAA-450, *G3U99_13155* from *V. coralliilyticus* OCN008, and *JV59_31975* from *V. coralliilyticus* OCN014 |
| tssM2_R_ForPCR | atggcagaatcaacttcatctaaatctacaaac |  |
| pBAD33.1^F^_Gib_ F | GATTACAAGGATGACGACGATAAGTGAAAGCTTGGCTGTTTTGGCGG | Used to amplify pBAD33.1^F^ and pKara1 plasmids backbone with a C-terminal FLAG tag for Gibson assembly |
| pBAD33.1^F^_Gib_ R | CATATGTATATCTCCTTCTTAAAGTTAAACAAAATTATTTCTAGAG |  |
| WP_006957348.1_pBAD33.1_F | CTTTAAGAAGGAGATATACATatgcgtccaacgaaagaaaacc | Used to amplify the CDS of WP_006957348.1 to construct pWP_006957348.1 |
| WP_006957348.1_pBAD33.1_R | CGTCGTCATCCTTGTAATCcttaaacgtccttaccgacaacac |  |
| WP_006959328.1_pBAD33.1_F | CTTTAAGAAGGAGATATACATatgactgatacattatcggcgtc | Used to amplify the CDS of WP_006959328.1 to construct pWP_006959328.1 |
| WP_006959328.1_pBAD33.1_R | CGTCGTCATCCTTGTAATCtttggaggcgggactc |  |
| WP_006959836.1_pBAD33.1_F | CTTTAAGAAGGAGATATACATatgtataaaaatgatgctattgc | Used to amplify the CDS of WP_006959836.1 to construct pWP_006959836.1 and pKara1:WP_006959836.1 |
| WP_006959836.1_pBAD33.1_R | CGTCGTCATCCTTGTAATCcaagaaatattctaacg |  |
| WP_006960006.1_pBAD33.1_F | CTTTAAGAAGGAGATATACATatgaaggacaaaattgttg | Used to amplify the CDS of WP_006960006.1 to construct pWP_006960006.1 |
| WP_006960006.1_pBAD33.1_R | CGTCGTCATCCTTGTAATCggctttagccac |  |
| WP_006961218.1_pBAD33.1_F | CTTTAAGAAGGAGATATACATatgtatgaatttttagtacgagac | Used to amplify the CDS of WP_006961218.1 to construct pWP_006961218.1 and pKara1:WP_006961218.1 |
| WP_006961218.1_pBAD33.1_R | CGTCGTCATCCTTGTAATCatctaacactaaacttc |  |
| WP_006961766.1_pBAD33.1_F | CTTTAAGAAGGAGATATACATatgattaccgattacgcaaaatatc | Used to amplify the CDS of WP_006961766.1 to construct pWP_006961766.1 |
| WP_006961766.1_pBAD33.1_R | CGTCGTCATCCTTGTAATCataatcctgataggcc |  |
| WP_006962231.1_pBAD33.1_F | CTTTAAGAAGGAGATATACATatgaaagtaaaaatcggggttac | Used to amplify the CDS of WP_006962231.1 to construct pWP_006962231.1 |
| WP_006962231.1_pBAD33.1_R | CGTCGTCATCCTTGTAATCgtttggtgactcttc |  |
| WP_006962597.1_pBAD33.1_F | CTTTAAGAAGGAGATATACATatgcaagtcaaactacttaaacgtc | Used to amplify the CDS of WP_006962597.1 to construct pWP_006962597.1 and pKara1:WP_006962597.1 |
| WP_006962597.1_pBAD33.1_R | CGTCGTCATCCTTGTAATCcacatccaatggtg |  |
| WP_141650583.1_pBAD33.1_F | CTTTAAGAAGGAGATATACATATGtcaaactttctgtttgtaggtg | Used to amplify the CDS of 141650583.1 to construct pWP_141650583.1 |
| WP_141650583.1_pBAD33.1_R | CGTCGTCATCCTTGTAATCtattttcaagcttctg |  |
| pGML10_Gib_F | AATTCTGAACAGAAACTGATTTCCGAAGAGGATCTG | Used to amplify the pGML10 plasmid backbone with a C-terminal Myc tag for Gibson assembly |
| pGML10_Gib_R | TCTAGAGTCAATTCGATCCGGGGT |  |
| pGML10:WP_006957348.1_F | CGGATCGAATTGACTCTAGAatgcgtccaacgaaagaaaac | Used to amplify the CDS of WP_006957348.1 to construct pGML10:WP_006957348.1 |
| pGML10:WP_006957348.1_R | GGAAATCAGTTTCTGTTCAGAATTcttaaacgtccttaccgacaac |  |
| pGML10:WP_006959328.1_F | ACCCCGGATCGAATTGACTCTAGAatgactgatacattatcggcg | Used to amplify the CDS of WP_006959328.1 to construct pGML10:WP_006959328.1 |
| pGML10:WP_006959328.1_R | GGAAATCAGTTTCTGTTCAGAATTtttggaggcgggactcg |  |
| pGML10:WP_006959836.1_F | ACCCCGGATCGAATTGACTCTAGAatgtataaaaatgatgctattg | Used to amplify the CDS of WP_006959836.1 to construct pGML10:WP_006959836.1 |
| pGML10:WP_006959836.1_R | GGAAATCAGTTTCTGTTCAGAATTcaagaaatattctaacggcgc |  |
| pGML10:WP_006960006.1_F | ACCCCGGATCGAATTGACTCTAGAatgaaggacaaaattgttgc | Used to amplify the CDS of WP_006960006.1 to construct pGML10:WP_006960006.1 |
| pGML10:WP_006960006.1_R | GGAAATCAGTTTCTGTTCAGAATTggctttagccaccgtctttac |  |
| pGML10:WP_006961218.1_F | ACCCCGGATCGAATTGACTCTAGAatgtatgaatttttagtacgag | Used to amplify the CDS of WP_006961218.1 to construct pGML10:WP_006961218.1 |
| pGML10:WP_006961218.1_R | GGAAATCAGTTTCTGTTCAGAATTatctaacactaaacttcgttg |  |
| pGML10:WP_006961766.1_F | ACCCCGGATCGAATTGACTCTAGAatgattaccgattacgcaaaatatc | Used to amplify the CDS of WP_006961766.1 to construct pGML10:WP_006961766.1 |
| pGML10:WP_006961766.1_R | GGAAATCAGTTTCTGTTCAGAATTataatcctgataggccccactttc |  |
| pGML10:WP_006962231.1_F | ACCCCGGATCGAATTGACTCTAGAatgaaagtaaaaatcggggttac | Used to amplify the CDS of WP_006962231.1 to construct pGML10:WP_006962231.1 |
| pGML10:WP_006962231.1_R | GGAAATCAGTTTCTGTTCAGAATTgtttggtgactcttccagttttaac |  |
| pGML10:WP_006962597.1_F | ACCCCGGATCGAATTGACTCTAGAatgcaagtcaaactacttaaac | Used to amplify the CDS of WP_006962597.1 to construct pGML10:WP_006962597.1 |
| pGML10:WP_006962597.1_R | GGAAATCAGTTTCTGTTCAGAATTcacatccaatggtggcatgcc |  |
| pGML10:WP_141650583.1_F | ACCCCGGATCGAATTGACTCTAGAatgtcaaactttctgtttgtag | Used to amplify the CDS of WP_141650583.1 to construct pGML10:WP_141650583.1 |
| pGML10:WP_141650583.1_R | TTTCTGTTCAGAATTtattttcaagcttctgggcacctcgtccatc |  |

^#^ Uppercase, plasmid sequence; lowercase, insert sequence

**Supplementary References**

1. Ben-Haim Y, Zicherman-Keren M, Rosenberg E. Temperature-regulated bleaching and lysis of the coral Pocillopora damicornis by the novel pathogen Vibrio coralliilyticus. Appl Environ Microbiol. 2003;69: 4236–42. Available: http://www.ncbi.nlm.nih.gov/pubmed/12839805

2. Kanarek K, Fridman CM, Bosis E, Salomon D. The RIX domain defines a class of polymorphic T6SS effectors and secreted adaptors. Nat Commun 2023 141. 2023;14: 1–13. doi:10.1038/s41467-023-40659-2

3. Ushijima B, Videau P, Burger AH, Shore-Maggio A, Runyon CM, Sudek M, et al. Vibrio coralliilyticus strain OCN008 is an etiological agent of acute montipora white syndrome. Appl Environ Microbiol. 2014;80. doi:10.1128/AEM.03463-13

4. Ushijima B, Videau P, Poscablo D, Vine V, Salcedo M, Aeby G, et al. Complete genome sequence of Vibrio coralliilyticus strain OCN014, isolated from a diseased coral at Palmyra Atoll. Genome Announc. 2014;2. doi:10.1128/genomeA.01318-14

5. Chung HS, Raetz CRH. Interchangeable domains in the Kdo transferases of escherichia coli and haemophilus influenzae. Biochemistry. 2010;49: 4126–4137. doi:10.1021/bi100343e

6. Fridman CM, Keppel K, Gerlic M, Bosis E, Salomon D. A comparative genomics methodology reveals a widespread family of membrane-disrupting T6SS effectors. Nat Commun. 2020;11: 1085. doi:10.1038/s41467-020-14951-4

7. Dar Y, Jana B, Bosis E, Salomon D. A binary effector module secreted by a type VI secretion system. EMBO Rep. 2022;23: e53981. doi:10.15252/embr.202153981

8. Dunn AK, Millikan DS, Adin DM, Bose JL, Stabb E V. New *rfp*- and pES213-derived tools for analyzing symbiotic *Vibrio fischeri* reveal patterns of infection and *lux* expression in situ. Appl Environ Microbiol. 2006;72: 802–810. doi:10.1128/AEM.72.1.802-810.2006

9. O’Toole R, Milton DL, Wolf-Watz H. Chemotactic motility is required for invasion of the host by the fish pathogen Vibrio anguillarum. Mol Microbiol. 1996;19: 625–637. doi:10.1046/j.1365-2958.1996.412927.x
