# Supplementary material for: The coral pathogen Vibrio coralliilyticus uses a T6SS to secrete a group of novel anti-eukaryotic effectors that contribute to virulence
Source: PLoS Biol. 2024 Sep 3;22(9):e3002734. doi: 10.1371/journal.pbio.3002734 (PMC11371242; doi:10.1371/journal.pbio.3002734)

**Fig. 2A**

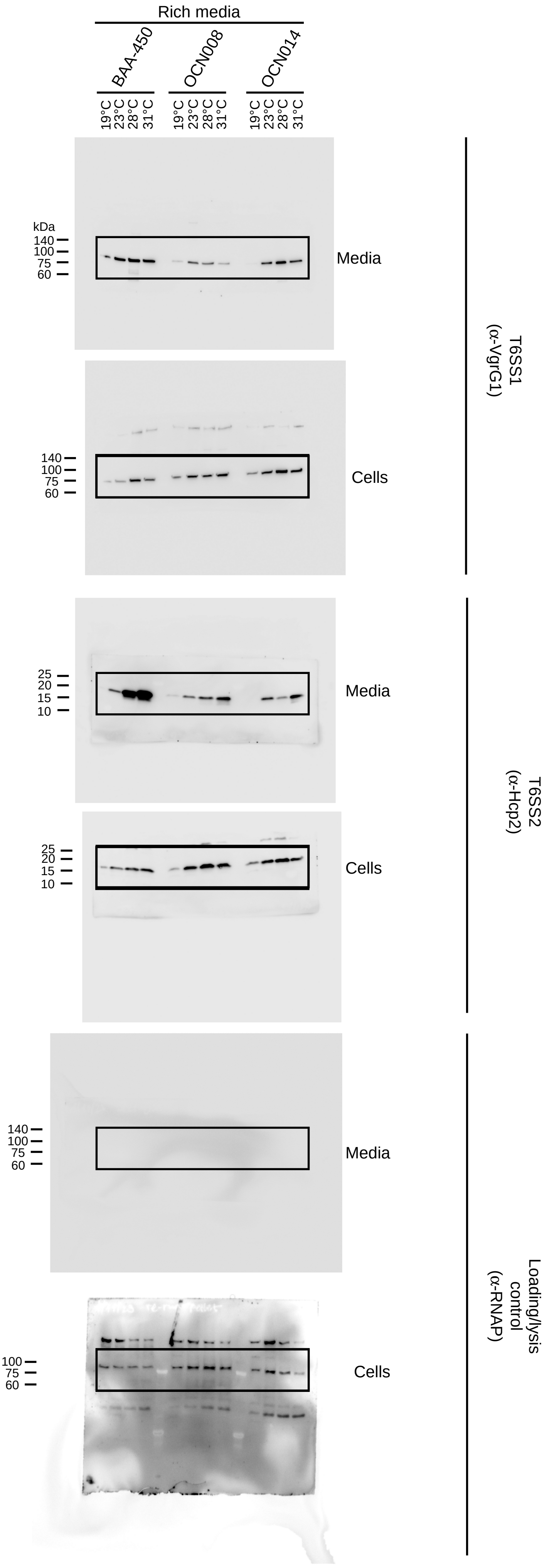

Rich media
BAA-450
OCN008
OCN014
19°C
23°C
28°C
31°C
kDa
140
100
75
60
Media
Cells
25
20
15
10
T6SS1
(α-VgrG1)
T6SS2
(α-Hcp2)
Loading/lysis
control
(α-RNAP)

**Fig. 2B**

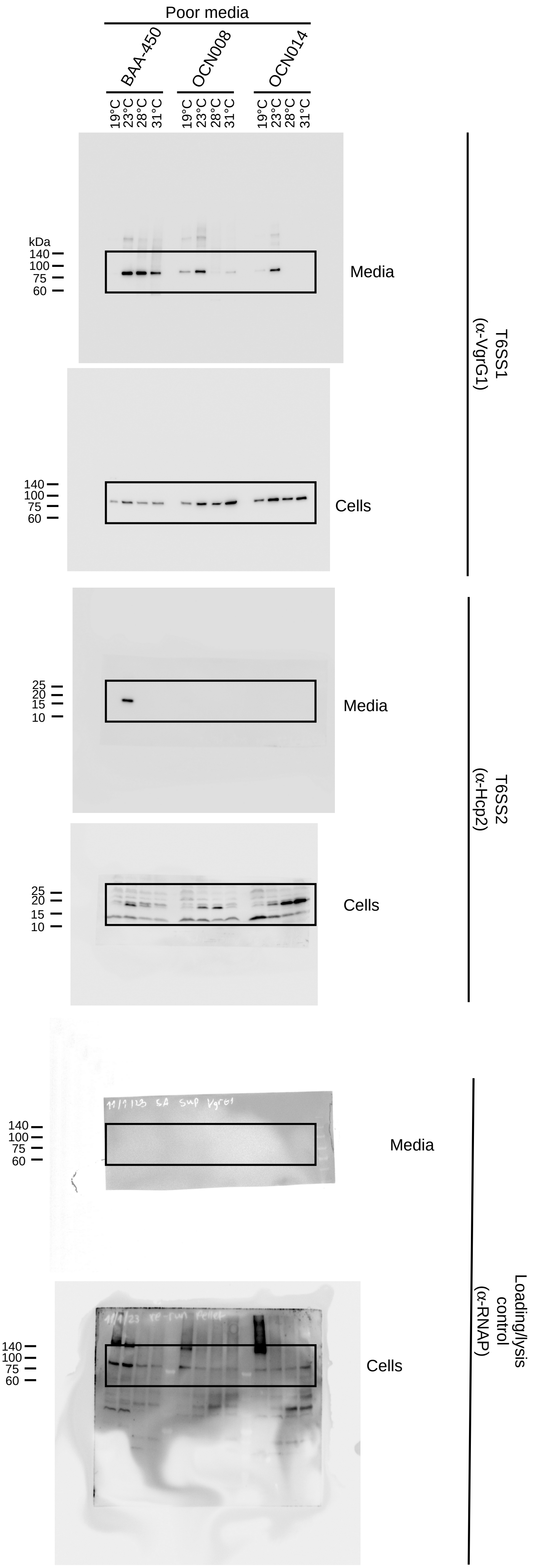
Poor media
BAA-450
OCN008
OCN014
19°C 23°C 28°C 31°C
19°C 23°C 28°C 31°C
19°C 23°C 28°C 31°C
kDa
140
100
75
60
Media
140
100
75
60
Cells
T6SS1 (α-VgrG1)
25
20
15
10
Media
25
20
15
10
Cells
T6SS2 (α-Hcp2)
140
100
75
60
Media
140
100
75
60
Cells
Loading/lysis control (α-RNAP)

**Fig. 2C**

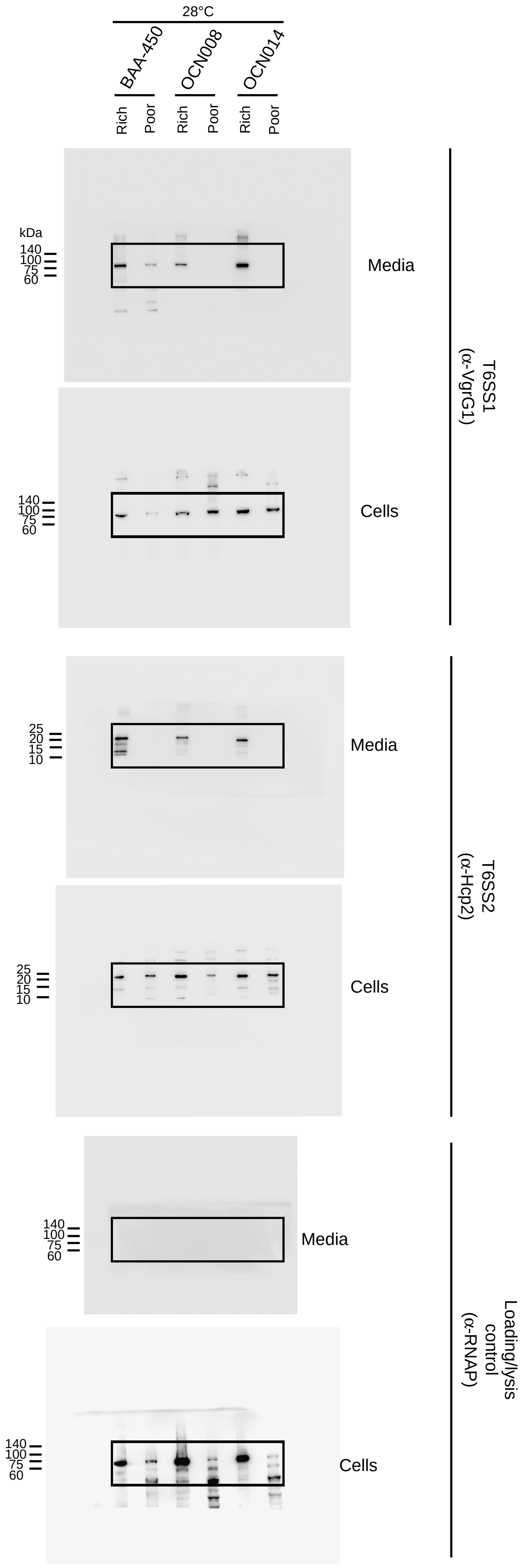
28°C
BAA-450
OCN008
OCN014
Rich
Poor
Rich
Poor
Rich
Poor
kDa
140
100
75
60
Media
Cells
T6SS1 (α-VgrG1)
25
20
15
10
T6SS2 (α-Hcp2)
Loading/lysis control (α-RNAP)

**Fig C in S1 Text**

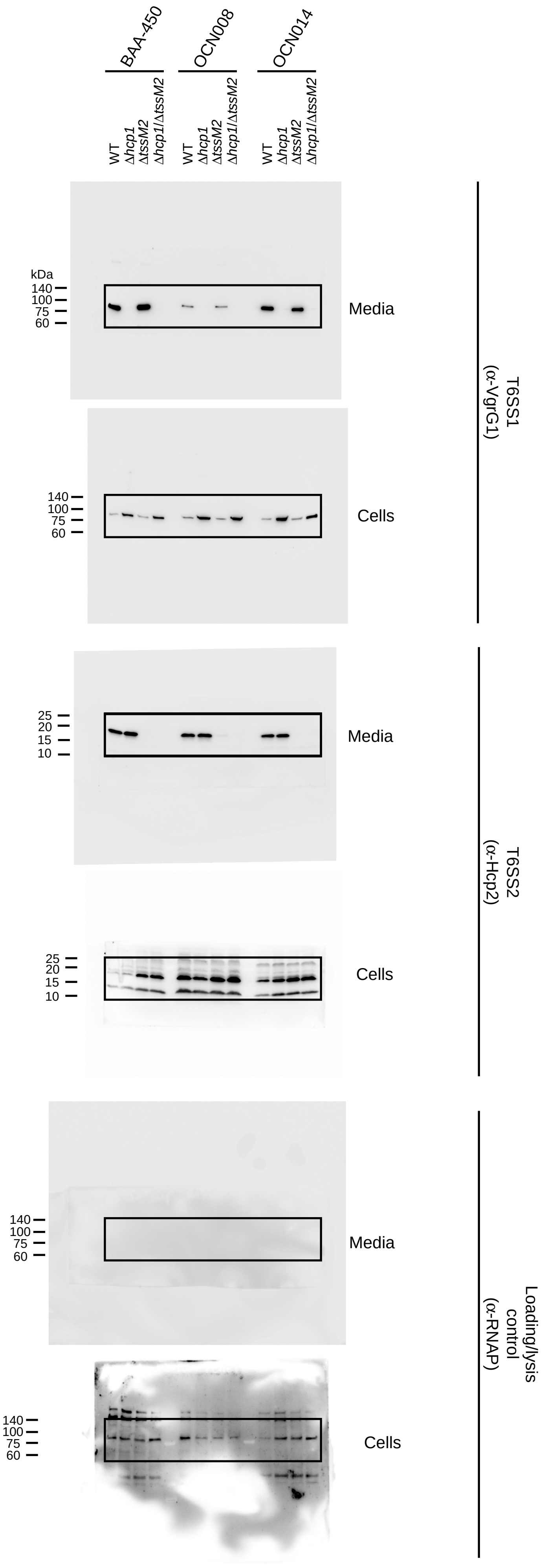

# Fig F in S1 Text

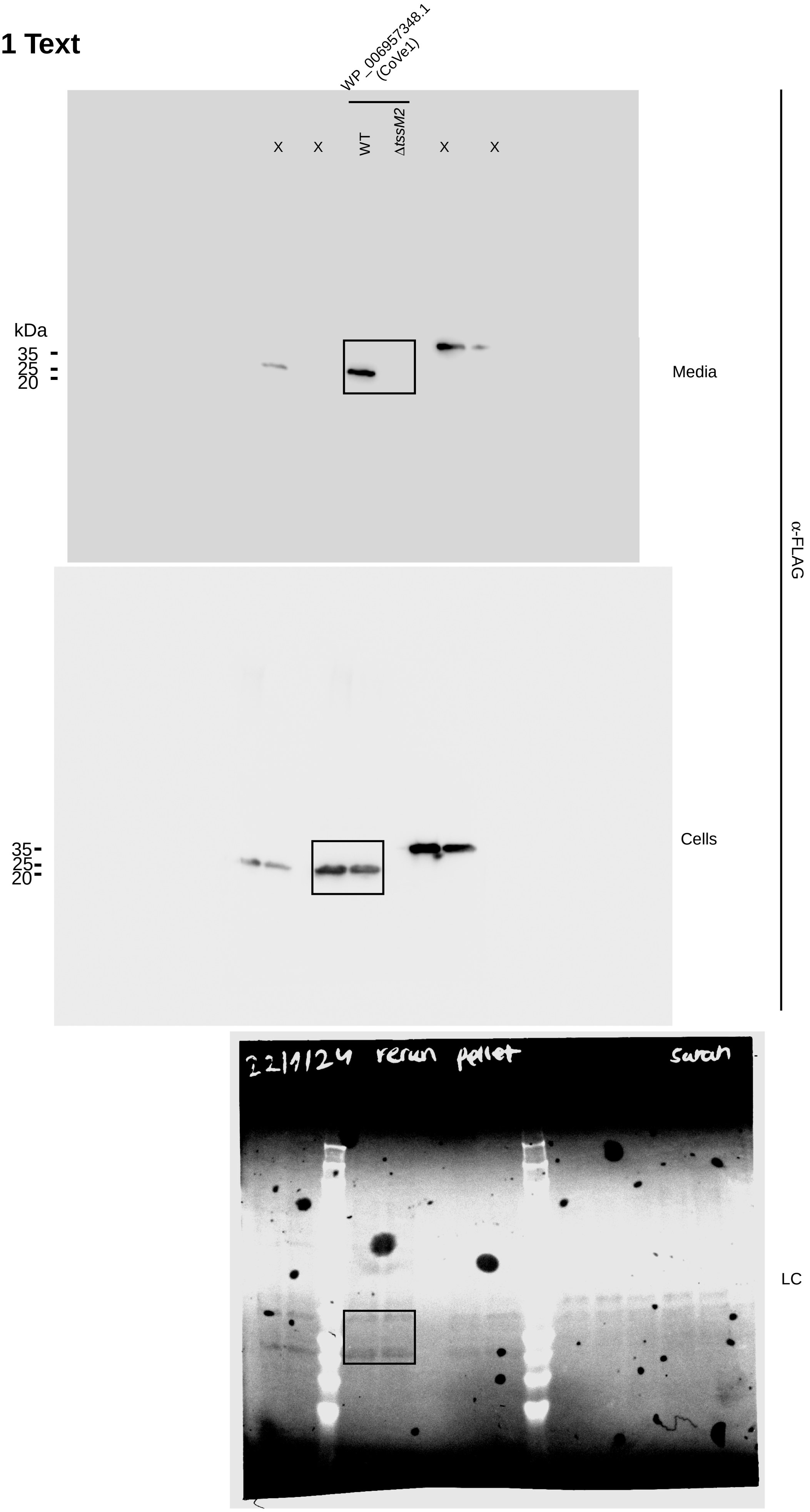

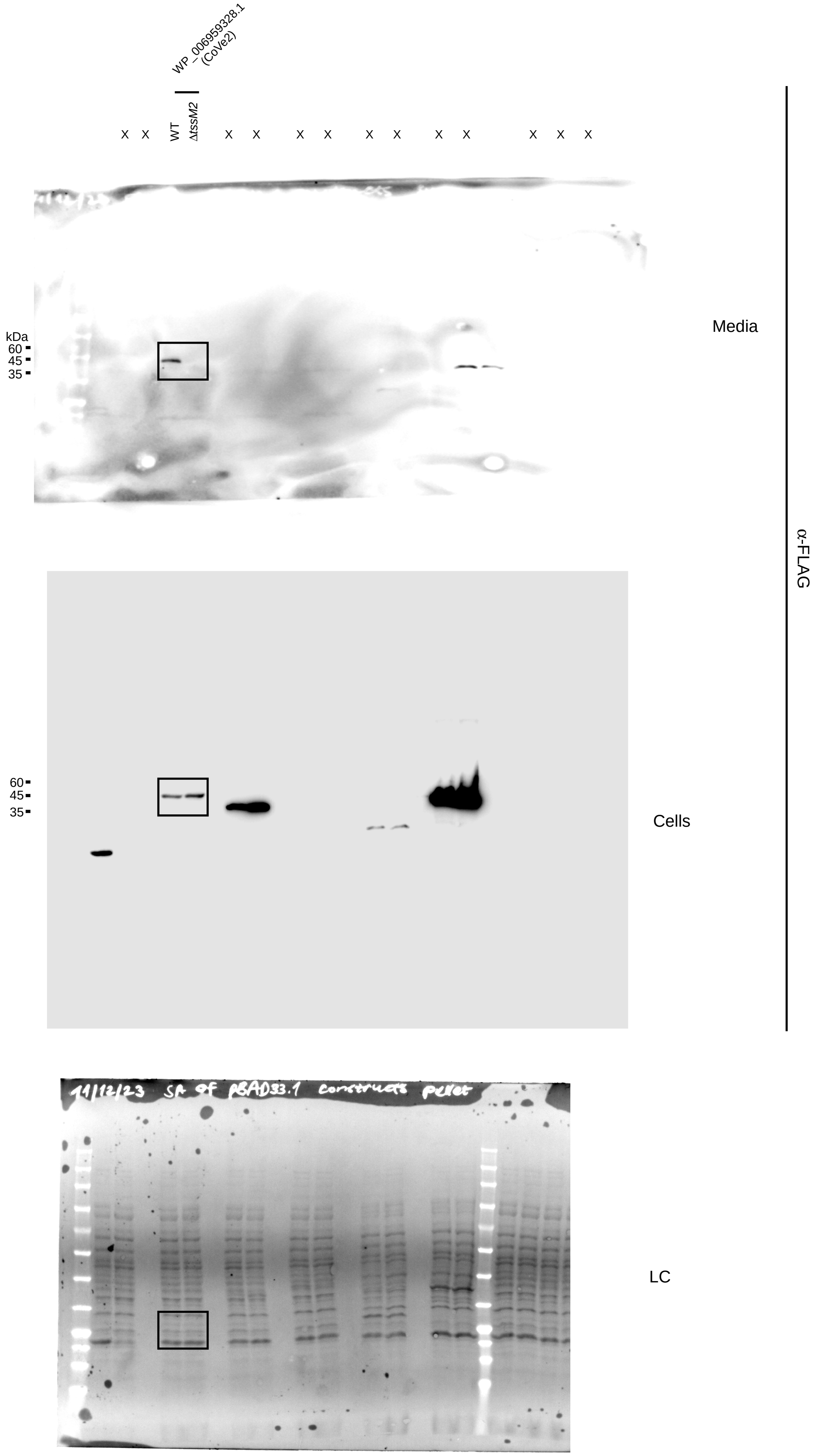
WP_006959328.1
(CoVe2)
WT
ΔtssM2
kDa
60
45
35
Media
Cells
LC
α-FLAG

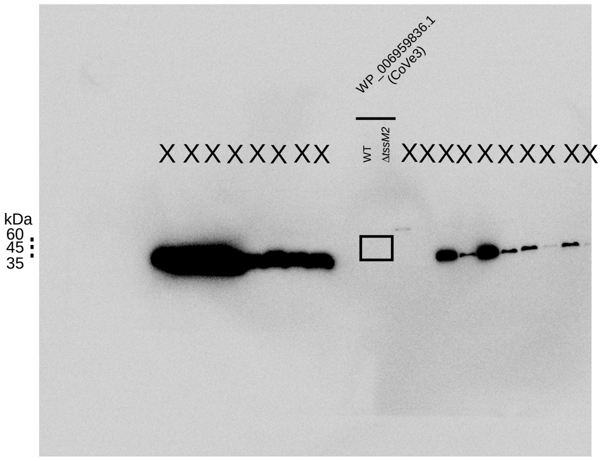


Media
(Strong exposure
of membrane below)

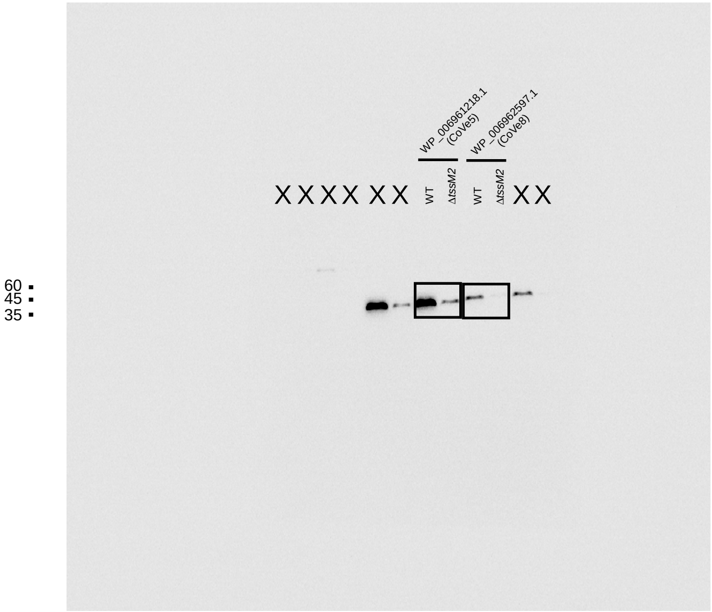


Media
(Short exposure of
membrane above after
removal of left side
before exposure)

α-FLAG

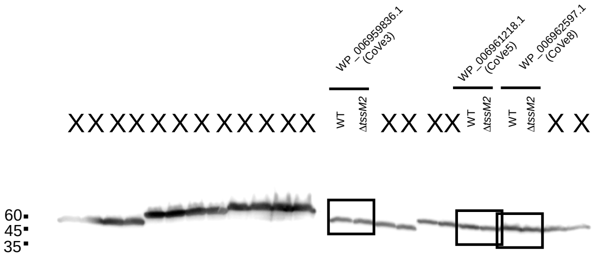


Cells

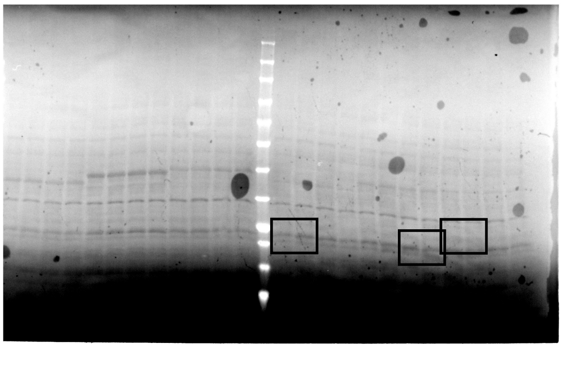

LC

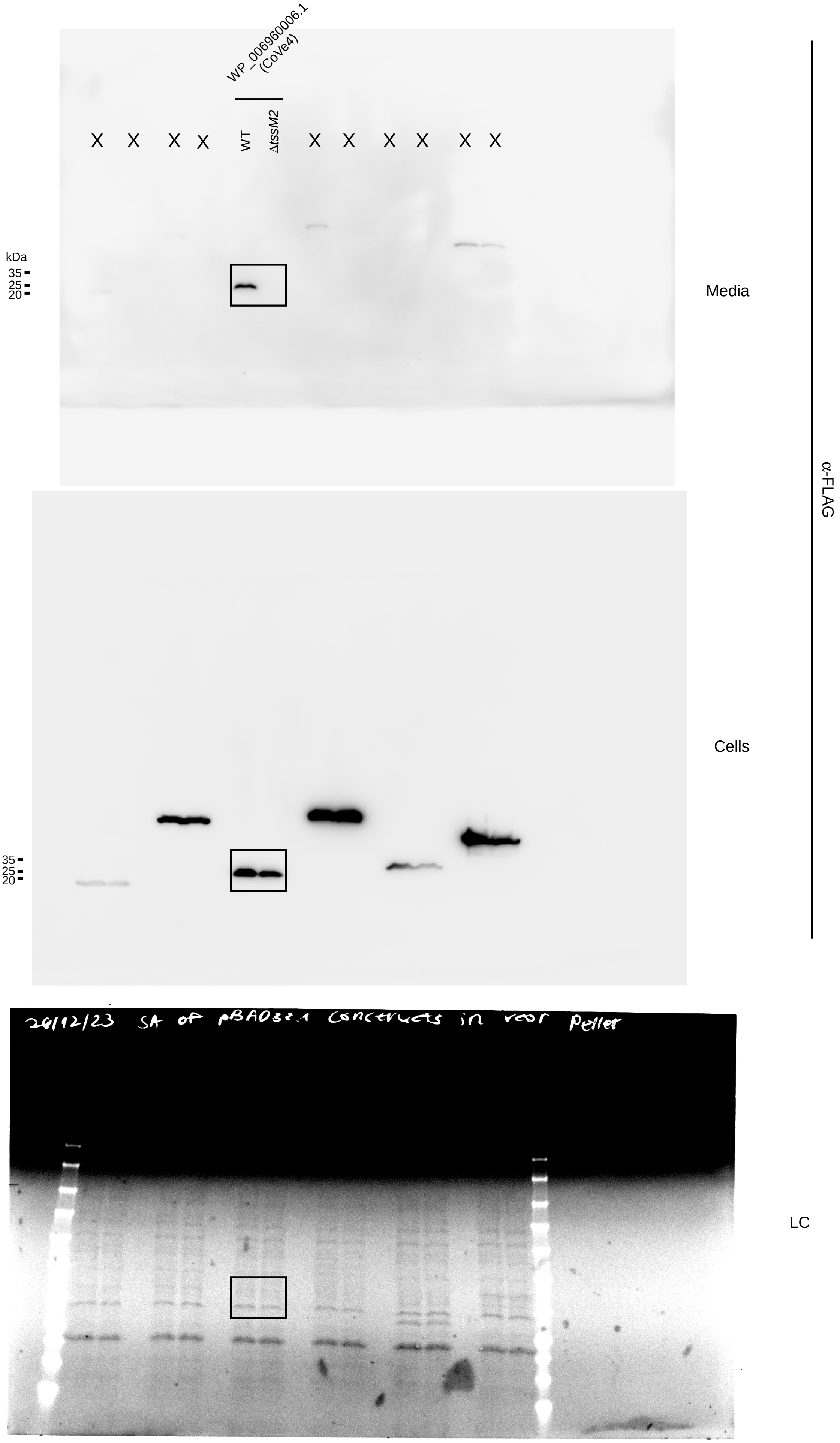
WP_006960006.1
(CoVe4)
X X X X WT ΔtssM2 X X X X X X
kDa
35
25
20
Media
35
25
20
Cells
α-FLAG
26/12/23 SA of pBAD33.1 Constructs in vcor Pellet
LC

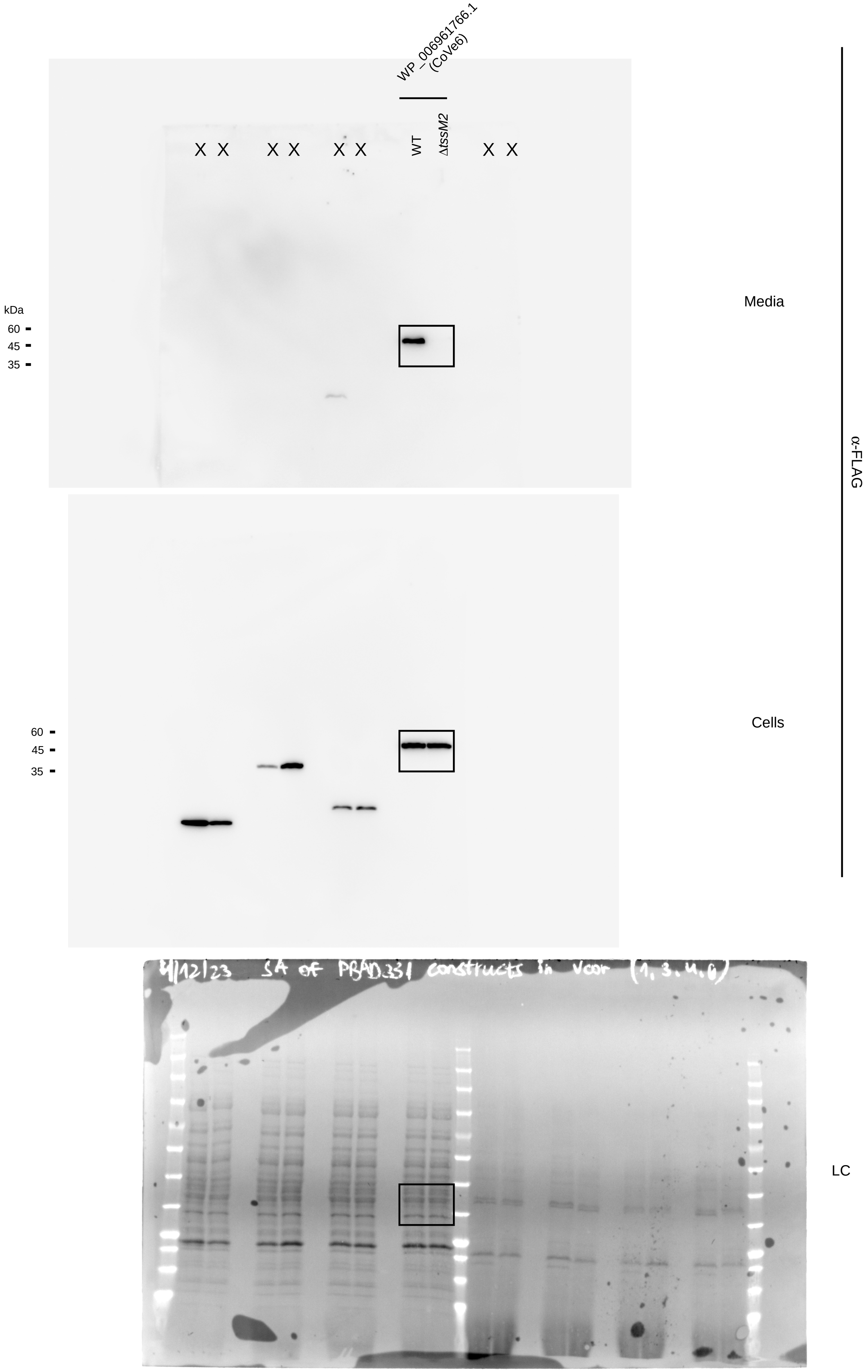
WP_006961766.1
(CoVe6)
X X
X X
X X
WT
ΔtssM2
X X
kDa
60
45
35
Media
60
45
35
Cells
α-FLAG
LC

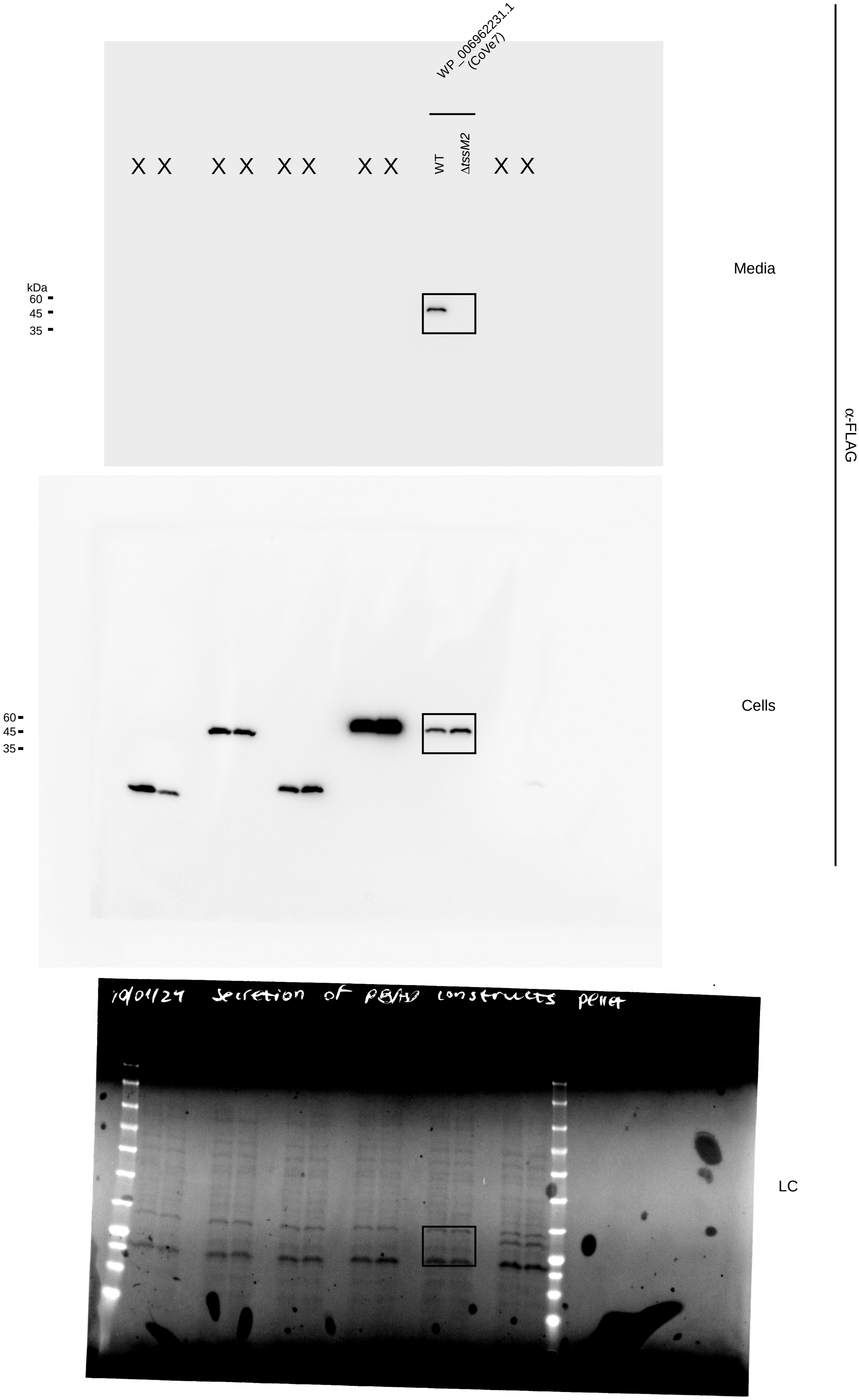
WP_006962231.1 (CoVe7)
X X X X X X X X WT ΔtssM2 X X
Media
kDa
60
45
35
Cells
60
45
35
LC
α-FLAG

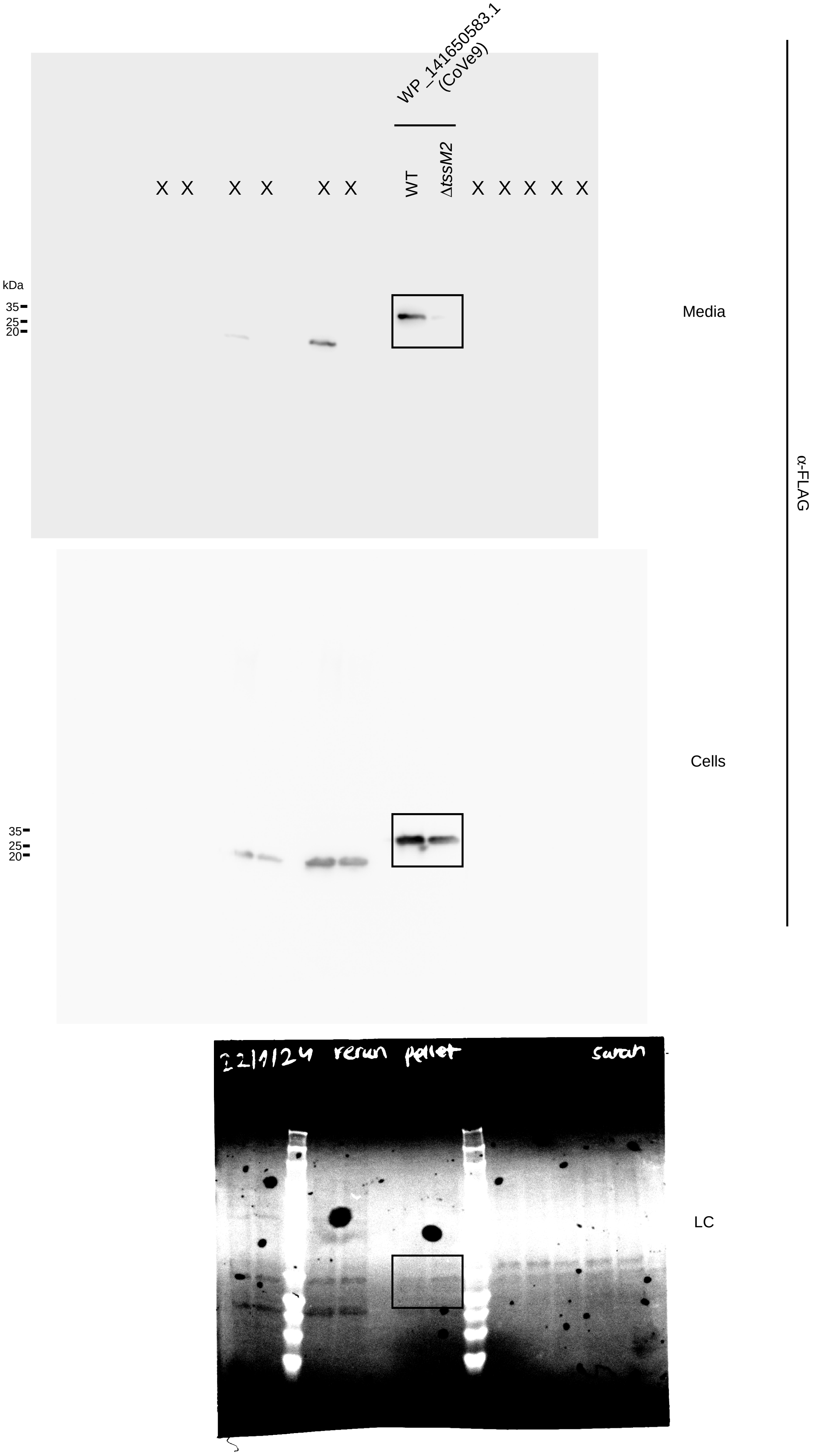
WP_141650583.1
(CoVe9)
WT
ΔtssM2
X X X X X X X X X X X
kDa
35
25
20
Media
Cells
LC
α-FLAG
22/11/24 rerun pellet
Sarah

# Fig G in S1 Text

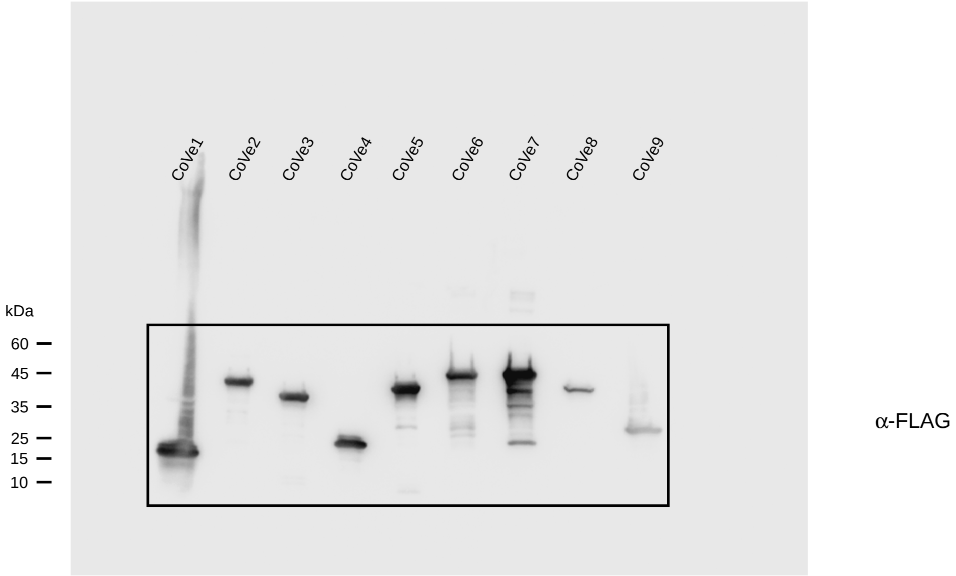


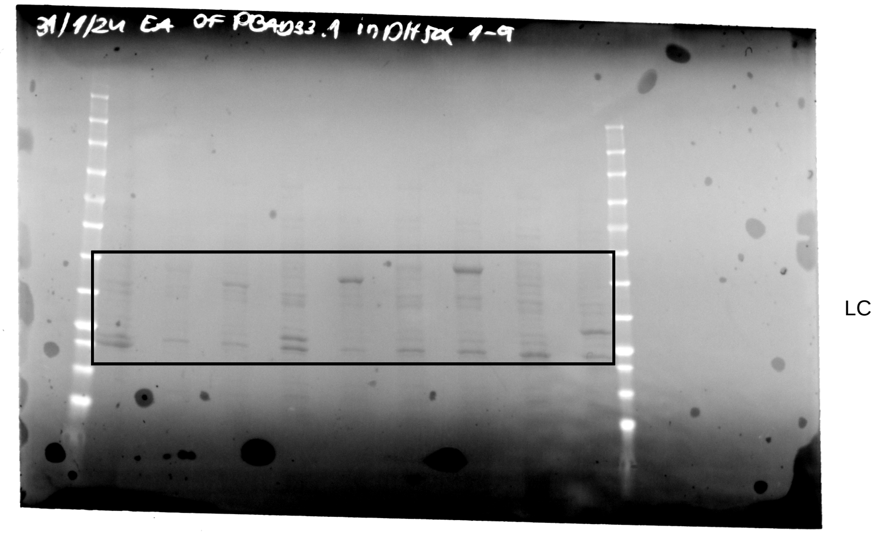

Supplement: S1 Raw Images — (PDF) [file pbio.3002734.s015.pdf]
