# Supplementary material for: The coral pathogen Vibrio coralliilyticus uses a T6SS to secrete a group of novel anti-eukaryotic effectors that contribute to virulence
Source: PLoS Biol. 2024 Sep 3;22(9):e3002734. doi: 10.1371/journal.pbio.3002734 (PMC11371242; doi:10.1371/journal.pbio.3002734)
Supplement: Attachment — Submitted filename: PBP.docx [file pbio.3002734.s016.docx]

**Point-by-point response to reviewers’ comments**

**Reviewer #1:**

The manuscript by Shir Mass et al., titled "A T6SS in the coral pathogen Vibrio coralliilyticus secretes an arsenal 2 of anti-eukaryotic effectors and contributes to virulence," analyzed the T6SS in Vibrio coralliilyticus and identified two omnipresent T6SS systems, T6SS1 and T6SS2. T6SS1 is involved in interbacterial competition, while T6SS2 mediates anti-eukaryotic toxicity and contributes to mortality during infection of Artemia salina. Through comparative proteomics, they identified the effectors of T6SS1 and T6SS2 in three Vcor strains, including nine novel effectors secreted by T6SS2. The results of this study are interesting and informative. Overall, it is a well-organized and well-written manuscript with rigorous logic.

**We thank the reviewer for the kind assessment of our work.**

There is a bit room for improvement and I have only two minor comments:

1. The only downside is the lack of coral infection assay mediated by T6SS2 effectors.

**We are working on establishing a coral facility to perform such infection assays. However, this is beyond the scope of the current manuscript.**

On line 122, the authors performed comparative proteomics analyses to reveal the Vcor T6SS secretomes and identify the effectors. If possible, the mutant strains by deleting genes encoding effectors like toxic proteins could be constructed and used to test the pathogenicity for Artemia salina.

**We agree that it would be interesting to determine whether specific effectors contribute more than others to virulence. However, we think that deciphering the specific activities and roles of the novel effectors is beyond the scope of this report.**

2. The authors competed Vcor strains against a sensitive a V. natriegens prey strain on rich media. It will be better if the selected vibrio strain is more representative or isolated in the natural environment, such as coral tissue, gastric cavity of shellfish larvae.

**Following the reviewer’s suggestion, we now show that *Vcor* also uses T6SS1 to outcompete *V. campbellii* and *V. alginolyticus* (Fig. S4), which are species previously isolated from corals (Alves et al., Environmental Microbiology, 2010): “The T6SS1-mediated killing was also apparent when *V. coralliilyticus* OCN008 was competed against *V. alginolyticus* or *V. campbellii*, two species that were previously isolated from corals”.**

BTW, on line 164, "a sensitive a V. natriegens" into "a sensitive V. natriegens".

**Thank you. The mistake was corrected.**

**Reviewer #2:**

This study presents compelling evidence that two Type VI Secretion Systems (T6SS) are conserved in Vibrio coralliilyticus, a pathogen affecting corals and oysters. Both T6SS systems are activated at ecologically relevant temperatures (28°C), yet they differ in their secretion profiles: T6SS1 targets prokaryotes, while T6SS2 secretes anti-eukaryotic effectors. Notably, T6SS2 induces mortality in artemia, serving as a model system for pathogenicity assessment. Through comparative proteomics, the authors identified nine novel putative toxins that specifically target eukaryotic cells, shedding light on the diversity of T6SS effectors.
Overall, the data presented are convincing and innovative, supported by a well-designed experimental approach. This study underscores the vast potential for uncovering new insights into T6SS dynamics using non-human Vibrio pathogens.

**We thank the reviewer for the kind assessment of our work.**

I only have minor comments

Introduction

The assertion regarding the spread of vibrios to new regions correlating with rising ocean temperatures might benefit from rephrasing to avoid potential misinterpretation. Indeed I disagree with this statement. Vibrios encompass a wide range of species inhabiting diverse environments, including psychrophiles. There is a tendency for publications to conflate human pathogens like V. cholerae and V. parahaemolyticus with the broader category of vibrios, because research historically focused heavily on V. cholerae.

**The reviewer’s point is well-taken. We rephrased the statement to clarify that it specifically describes human pathogens: “In the past, human pathogenic vibrios were primarily associated with the warmer equatorial waters”.**

Additionally, please use "V. coralliilyticus" instead of "Vcor" everywhere

***Vcor* was replaced with *V. coralliilyticus* throughout the text.**

References to Rubio et al. (PNAS, 2019) and works from the Blokesch lab, particularly Drebes Dörr et al. (Environmental Microbiology, 2020), should be included for comprehensive coverage.

**Thank you for suggesting these references. We added them to the introduction.**

Results:

Consider incorporating a figure depicting the core genome phylogeny of the 31 V. coralliilyticus strains and the synteny of T6SS1 and T6SS2 to facilitate data interpretation.

**The core genome phylogeny of the 31 strains is now shown in Fig. 8. The synteny of T6SS1 and T6SS2 is similar in all 31 strains, as is now shown in Fig. S1 and S2.**

Clarify the term "Marine LB" (Line 134) for improved understanding.

**We now specify that this means LB containing 3% (wt/vol) NaCl.**

Regarding the comparison of T6SS activity between rich and poor media at 28°C (Line 142), adjust the statement to reflect the relative protein abundance differences more accurately (clearly less proteins in poor media).

**The sentence was revised to: “Comparison between the activity of both systems in rich and poor media at 28°C revealed higher levels of expression and secretion in rich media”.**

For Figures 4B, C, D, provide clarification on the statistical significance of the results, particularly regarding the differences between T6SS1 and T6SS2 mutants.
It appears that for one strain T6SS1 has an effect (linked to MIX domain-containing effectors?)

**We added statistical analyses of the BMDM infection results (Fig. 4). Indeed, for strain BAA-450, there is a statistically significant difference not only between the WT and the *tssM2* mutants but also between the WT and the *hcp1* mutant. This difference could result, as suggested by the reviewer, from the effect of the predicted anti-eukaryotic MIX effector found in this strain. We now mention this in the Discussion section: “Notably, we observed a mild effect of T6SS1 inactivation on the toxicity of the BAA-450 strain toward BMDMs, suggesting that T6SS1 may exert some effect also on eukaryotic cells. This could be mediated by the anti-eukaryotic MIX effector identified in the comparative proteomics analysis. However, we did not observe a similar effect with strains OCN008 and OCN014, even though they, too, secrete a predicted anti-eukaryotic MIX effector (Table 1)”.**

In Figure 8, consider presenting a core genome phylogeny and include bootstrap values for clarity.

**The *rpoB*-based phylogenetic tree in Fig. 8 was replaced by a core-genome phylogenetic tree (based on 1,210 core proteins) including bootstrap values, as suggested.**

Further clarification is needed regarding the rationale behind selecting strains for phylogeny in Figure 8 and the presence of homologs of CoVe2 and CoVe8 in all V. coralliilyticus genomes. Do they mean paralog/gene duplication?

**We mention in the relevant results section that “we retrieved the sequences of the core T6SS sheath component, TssB, from 31 available RefSeq *V. coralliilyticus* genomes”.**

**This is now further elaborated in the Methods section: “*V. coralliilyticus* genomes under NCBI Taxonomy ID=190893 were retrieved from the local database…”**

**Regarding the homologs of CoVe2 and CoVe8 – at this time, we lack sufficient data to determine whether these are the result of gene duplication. Therefore, we prefer not to elaborate beyond the current description.**

Consideration could be given to testing the deletion of candidate toxins in cellular assays, such as BMDM, for a more comprehensive understanding of their role in pathogenicity. If they tried but did not get an effect, what does it mean?

**We agree that it would be interesting to determine whether specific effectors contribute more than others to virulence. However, we think that deciphering the specific activities of the novel effectors is beyond the scope of this report.**

Discussion

Reevaluate the necessity of certain paragraphs, such as lines 355-364, for enhanced coherence and relevance.

**The paragraph was removed.**

Line 380: Ensure clarity in statements regarding the nature of Artemia infection assays. I don't believe V. coralliilyticus kills artemia externally, but rather after being ingested. Consequently, the bacteria are not exposed to ocean salt but instead to the stomach environment, which may not be nutrient-poor.

**Indeed, the virulence toward *Artemia* likely originates in the animal gut. We have revised the paragraph for clarity: “*Artemia* nauplii were grown in poor media (i.e., Instant Ocean), yet the infection likely originated in the gastrointestinal tract of the animal, where nutrients may be more readily available. Host factors found in the animal gut may have also contributed to the activation of the T6SS during infection”.**

Lines 387-389. What is meant here? Is it suggesting that V. coralliilyticus could be a human pathogen? This assertion seems somewhat speculative and should not be the concluding statement. However, I find it intriguing that they conducted cellular assays with mouse cells, considering that Vibrio species from marine environments (non-human pathogen) typically struggle in salt-free media.

**We removed this hypothesis from the manuscript. We now state that the results obtained using the macrophages indicate “that *V. coralliilyticus* T6SS2 is functional under a wide temperature range, and can overcome immune cells”.**

**We think that the bacteria are quickly phagocytosed by the macrophages and thus are not exposed to the low salinity medium for long. Moreover, we and others (e.g., Cohen et al., eLife, 2022 and Ma et al., Cell Host & Microbe, 2009) previously showed that anti-eukaryotic *Vibrio* T6SSs often require engulfment by the host cell for effector delivery.**

Provide a stronger conclusion that encapsulates the main findings and implications of the study.

**We added a new summary paragraph at the end of the Discussion section: “In conclusion, we find a new T6SS that specifically targets eukaryotes, and we identify its effector arsenal. Taken together with recent findings of vibrios that use T6SSs to target eukaryotes of widespread *Vibrio*-encoded anti-eukaryotic T6SS effectors, our results suggest that T6SSs should be considered as potential virulence factors. These findings shed light on the molecular mechanisms that govern the connection between rising seawater temperatures and *V. coralliilyticus* virulence”.**

Reviewer #3:

The authors report studies on the role of the Type 6 Secretion Systems (T6SSs) of the bacterial organism Vibrio coralliilyticus (Vcor) in assays for anrti-bacterial, anti-eukaryotic phagocytic cells, and anti-higher organisms (Artemia salina or brine shrimp).  Because Vcor is a significant pathogen of both coral and shellfish, this study is potentially of high importance to understanding and potentially controlling this aquatic bacterial pathogen.   

The investigators performed studies to address whether the expression of two conserved ("omipresent') T6SSs of Vcor were regulated by growth temperature and found that indeed these were induced by elevated temperatures --- conditions that make Vcor particularly pathogenic for the model organism Artemia salina.   This result is important because previous studies have proposed that elevated ocean temperatures modulate the virulence of this organism particularly for coral in the context of bleaching disease. 

The investigators go on to convincingly show that the T6SS1 has a major role as an antibacterial weapon using standard co-incubation on agar assays.  They then show that the T6SS2 can elicit toxicity in both shrimp and in bone marrow derived macrophages from mice.  These anti-eukaryotic effects of the T6SS are relatively rare in the field and thus these are important new results.   The toxicity observed also correlated with loss of viability of the shrimp in their aquatic animal model.  Using state-of-the-art proteomics the authors also determined the array of proteins that were likely secreted by the T6SS machines in Vcor.  These included the usual structural components like Hcp, VgrG, and PAAR proteins but also a variety of likely effector proteins based on their homology to T6SS effector in other bacterial systems.  Several of these proteins also did not display homology to known effectors and thus may indeed be novel toxins for prokaryotic or eukaryotic cells. 

I found the manuscript well-written and now reasonable to accept for publication in PLOS Biology.  It addresses an important problem in microbiology -- how rising sea temperatures might affect the fitness and virulence of aquatic bacterial that in turn can then cause major economic and ecological damage globally.

**We thank the reviewer for the kind assessment of our work.**
